# Effect of a Trauma-Awareness Course on Teachers’ Perceptions of Conflict With Preschool-Aged Children From Low-Income Urban Households: A Cluster Randomized Clinical Trial

**DOI:** 10.1001/jamanetworkopen.2019.3193

**Published:** 2019-04-26

**Authors:** Robert C. Whitaker, Allison N. Herman, Tracy Dearth-Wesley, Hannah G. Smith, Samuel B. Burnim, Ellen L. Myers, Allison M. Saunders, Kirsten Kainz

**Affiliations:** 1 Columbia-Bassett Program, Vagelos College of Physicians and Surgeons, Columbia University, New York, New York; 2 Columbia-Bassett Program, Bassett Medical Center, Cooperstown, New York; 3 Bassett Research Institute, Bassett Medical Center, Cooperstown, New York; 4 Previously affiliated with Department of Epidemiology and Biostatistics, Center for Obesity Research and Education, College of Public Health, Temple University, Philadelphia, Pennsylvania; 5 Now affiliated with College of Medicine, State University of New York Upstate Medical University, Syracuse; 6 School of Social Work, University of North Carolina, Chapel Hill

## Abstract

**Importance:**

Adverse childhood experiences are common and are associated with changes in early development and learning, but training early childhood educators in trauma-informed approaches to care has not been evaluated with randomized clinical trials.

**Objective:**

To determine whether a 6-session (12-week) professional development course, “Enhancing Trauma Awareness,” improved the quality of teachers’ relationships with the children in their classrooms.

**Design, Setting, and Participants:**

This cluster randomized clinical trial conducted from September 2017 to May 2018 allocated classrooms by a computer-generated random sequence to intervention (attend Enhancing Trauma Awareness course) and control (no course) groups. Outcomes were reported by participants via survey and analyzed by group allocation. Classrooms under the auspice of the School District of Philadelphia, Pennsylvania, serving 3- and 4-year-old children living in low-income households were invited to participate. Lead and/or assistant teachers from 63 of 348 eligible classrooms (18.1%) agreed to participate, and none were excluded. Of 96 enrolled teachers, 93 (96.9%) were assessed at follow-up (61 of 63 classrooms [96.8%]).

**Exposures:**

In September 2017, 32 classrooms (48 teachers) were assigned to receive a professional development course that taught about the effects of trauma using a group-based relational process, and 31 classrooms (48 teachers) received no intervention. Teachers completed online surveys immediately before and after the course. Exploratory focus groups with intervention teachers (n = 15) were conducted 5 months after the course ended.

**Main Outcomes and Measures:**

The primary outcome was teacher-children relationship quality, with a hypothesized decrease in teacher-children conflict scores. Secondary outcomes included relational capacities (eg, empathy, emotion regulation, and dispositional mindfulness). Focus group themes described teachers’ experience of the course.

**Results:**

Of 96 teachers enrolled, 93 (96.9%) were women, and 58 (60.4%) were 40 years and older. Follow-up surveys were completed by 46 teachers (95.8%) in the control group and 47 (97.9%) in the intervention group, of whom 38 (79.2%) attended 4 or more course sessions. Adjusting for baseline values, mean (SE) conflict scores after the course were not significantly different between course participants (15.8 [0.6]) and controls (15.0 [0.6]) (effect size = 0.16; 95% CI, −0.19 to 0.52). There were no significant between-group differences in secondary outcomes. However, in focus groups, the teachers reported improvements in teacher-children relationship quality and several related relational capacities.

**Conclusions and Relevance:**

A course to enhance trauma awareness among preschool teachers did not reduce teacher-children conflict scores, yet qualitative assessments suggested the potential for improved teacher-children relationship quality.

**Trial Registration:**

ClinicalTrials.gov identifier: NCT03303482

## Key Points

**Question:**

Does a 6-session professional development course on trauma-informed care improve the quality of the relationships between early childhood teachers and children in their classrooms?

**Findings:**

A cluster randomized clinical trial with 96 teachers found that a course on trauma-informed care did not reduce teachers’ perception of conflict with preschool-aged children. However, in focus group interviews, teachers’ reports suggested improvements in the quality of teacher-children relationships and teachers’ relational capacities, such as empathy, emotion regulation, and dispositional mindfulness.

**Meaning:**

The course had no effect on the primary outcome of teacher-children conflict, yet qualitative assessments suggested the potential for improved teacher-children relationship quality.

## Teaser

This cluster randomized clinical trial determines whether a 6-session professional development course improves the quality of teachers’ relationships with the children in their classrooms vs teacher-children relationships in classrooms that did not receive the intervention.

## Introduction

Life events or circumstances can be considered traumas when they are experienced by an individual as harmful or threatening and have lasting adverse effects on functioning and well-being.^1^ Many adverse childhood experiences (ACEs), such as abuse and neglect, can be traumatic.^2^ Trauma-informed care, whether delivered through health care, human services, or education, refers to supportive ways of engaging people who may have had traumatic experiences.^1^ A broad range of activities can be considered part of trauma-informed care,^3^ and training and workforce development are integral parts of implementing such care.^1^ However, evidence is lacking about effective approaches to staff training. A review of the literature published since 2000 identified fewer than 25 evaluations of trauma-informed organizational interventions that included staff trainings.^4^ Only 4 of these evaluations were randomized clinical trials. Limited inferences could be made about the impact of staff trainings because the evaluations often involved questionnaires developed by those who created the trainings, had only short-term follow-up, used single-group designs, and/or could not separate the effects of staff training from other concurrent organizational interventions.

Much is understood about ACEs—their lifelong impacts on health and functioning,^5,6^ the sociobiologic mechanisms of these impacts,^7,8^ the societal costs,^9^ the intergenerational transmission,^10,11^ and the disproportionate burden on children living in poverty.^12^ These observations suggest that early childhood education programs serving low-income children may be a strategic place to implement trauma-informed approaches that could benefit children, families, and staff. Preschool-aged children experiencing trauma have impaired self-regulation, negative emotions, and disruptive behaviors that can interfere with learning and, in turn, later health.^13^ To develop the self-regulation needed for learning, children must have safe, stable, and nurturing relationships with adults,^14^ and to form these relationships, these adults must understand the role of trauma in their lives and the lives of children.^15^ The changes in knowledge, attitudes, and behaviors that may arise from professional development courses to increase trauma awareness could contribute to a greater sense of safety in the relationship between teacher and child.^16,17^ Such relational safety may also increase the effectiveness of interactions between social service professionals^18^ and clients as well as between health care professionals and patients.^3^

We independently evaluated the effects of a professional development course on trauma-informed care among preschool teachers in classrooms serving children living in low-income, urban households. The course was hypothesized to increase the quality of relationships between teachers and the children as measured by a reduction in perceived conflict between them. Secondary outcomes included changes in teachers’ reports of their relational capacities and health and well-being.

## Methods

In this cluster randomized clinical trial, each cluster was a preschool classroom, which was the unit of random assignment. Each cluster consisted of up to 2 persons—the lead teacher, the assistant teacher, or both, depending on who agreed to participate. A cluster design was chosen because the classroom lead and assistant teachers were intended to learn and interact as a pair during the professional development course being evaluated. This design supported a common structure in preschool classrooms in which 2 teachers work as partners in their daily interactions with children and parents. In September 2017, classrooms were randomly allocated, in a 1:1 ratio to the intervention group (Enhancing Trauma Awareness [ETA] course) or to the control group (no course). In February 2018, after the intervention period, teachers in the control group were offered ETA.

The primary effect of the intervention was assessed with data from online surveys of the teachers, and these outcomes were analyzed at the level of the teacher and accounted for clustering. No data were collected from children. In exploratory analyses, we used qualitative data from focus groups to understand how the course was experienced by the teachers who took it and the trainers who delivered it. The study protocol was approved by the institutional review boards of Temple University and the Mary Imogene Bassett Hospital as well as the Research Review Committee of the School District of Philadelphia, Pennsylvania. Written informed consent to participate in the trial was obtained from individual teachers before random assignment of classrooms. This study followed the Consolidated Standards of Reporting Trials (CONSORT) reporting guideline. The trial protocol is available in Supplement 1.

Preschool classrooms, under the auspice of the School District of Philadelphia, were eligible if they were located in centers (sites) that served 3- and 4-year-old children, exclusively funded by the federal Head Start program (income eligibility <100% of the federal poverty level) or the Pennsylvania Pre-K Counts program (income eligibility ≤300% of the federal poverty level). There were 348 eligible classrooms (696 teachers) across 133 sites. Each classroom had 20 children. We planned to enroll a single classroom per site with the lead and assistant teachers from that classroom; however, to achieve adequate sample size, teachers were permitted to enroll alone, and sites were permitted to enroll more than 1 classroom. Although 26 sites enrolled more than 1 classroom, only 8 sites had teachers in both the intervention and control groups after random assignment.

Given the availability of course trainers, up to 128 teachers could be recruited to the trial. This number was estimated to be sufficient to detect moderate mean differences in outcomes between intervention and control groups (effect size = 0.50), assuming 2 teachers per cluster, an intracluster correlation coefficient of 0.20,^19^ baseline variation in the outcome measure accounting for 50% of the variation at follow-up, an α of .05, and a power of 80%.

During the first 3 weeks of the 2017 to 2018 school year, all eligible teachers were notified about the study by their supervisors. Interested teachers visited a website to complete consent and recruitment. The study coordinator (A.N.H.) enrolled classrooms and notified teachers of their random group assignment, but the assignment was made by the study analyst (T.D.-W.). The coordinator submitted identification numbers of consecutively enrolled classrooms to the analyst in batches of 2 or more. Using Stata version 13 (StataCorp), the analyst assigned a randomly generated number to each classroom and placed classrooms in each batch in descending order of their random number. Classrooms on this list were assigned alternating values X or Y, reordered by classroom identification number, and returned to the coordinator. The analyst remained masked to which letter designated the intervention group.

### Intervention

The ETA course content, process, trainer preparation, and quality control are described in eAppendix 1 in Supplement 2. The course was taught in 6 sessions over the 12-week intervention period (2.5 hours every other week). The course was offered at 4 locations, with groups ranging in size from 9 to 16 teachers. Teachers were allowed to take the course at the most convenient location but were required to be in the same location as their coteacher and attend all sessions with the same group. There were 9 course trainers (2 assigned to each location and 1 substitute).

The course content, based on current theories of trauma and recovery,^17,20,21,22,23,24,25^ provided knowledge about the nature of trauma and how it can affect people’s emotions, behaviors, and biology. The course also taught basic skills for responding in a helpful manner to those who might be affected by trauma. Enhancing Trauma Awareness was designed to have a relational process based on the theories and techniques of group process,^26^ active learning,^27^ and adult learning,^28^ allowing participants to reflect on and apply the knowledge acquired. The trainers created a supportive emotional climate to help participants feel safe, respected, and nurtured. To preserve that climate, the evaluators did not directly observe or videotape ETA sessions. Attendance was recorded by the trainers.

### Primary Outcome Evaluation

Data were obtained from validated self-report items and instruments compiled into an online survey that was repeated for all teachers at 3 times: (1) baseline (late September to early October 2017, immediately before the intervention), (2) follow-up (December 2017, immediately after the intervention), and (3) delayed follow-up (early May 2018, 5 months after the end of the fall intervention and immediately after control teachers completed the course). Selection of survey measures was guided by a conceptual framework (eFigure in Supplement 2) with 3 outcome domains (relational capacities, health and well-being, and relationship quality) covering 12 constructs. The prespecified primary outcome was teachers’ perception of conflict in their relationships with children in their classrooms. We hypothesized that following the intervention, teacher-children conflict, a construct in the domain of relationship quality, would be lower in the intervention group than in the control group. The teacher-children conflict score was based on an 8-item scale adapted from the Student-Teacher Relationship Scale, Short Form^29,30^ (eTable 1 in Supplement 2). This modified version of the conflict scale required teachers to reflect on their classroom as a whole rather than selecting a particular child in the class.^30^ There were 21 additional prespecified secondary outcomes (eTable 1 in Supplement 2) measured across the remaining 11 constructs. Sociodemographic data were collected in an online survey at enrollment that also included 4 measures of economic hardship, described elsewhere.^30^ The teachers answered items in the baseline survey about whether they had exposure to ACEs.^31,32,33^

### Statistical Analysis

Outcomes were analyzed according to group allocation using Stata version 13 (StataCorp). Linear and logistic mixed-effects models were used to examine intervention and control group differences in the outcomes. For each model, the covariance structure was independent, and the unit of analysis was the teacher, with 2 nested levels of clustering (classrooms and sites). The fixed effects in the model were study group and the baseline measure for the outcome of interest. For outcomes, adjusted mean differences between the intervention and control group at follow-up were determined from linear models, while standardized prevalence differences at follow-up were determined from logistic models. Effect-size measures were derived in linear models^34^ and in logistic models from odds ratios. A significance threshold of *P* < .05 from 2-sided testing was used.

### Exploratory Focus Group Evaluation

The focus groups were exploratory and planned after the intervention began, when additional evaluation resources became available. We used these data to explore effects that may not have been adequately captured by closed-ended survey questions, especially intrapersonal and interpersonal processes and outcomes, and to assess the fidelity of intervention implementation. Three 90-minute focus groups were held approximately 5 months after the intervention: 2 involving 15 teachers in the intervention group, and 1 involving all 9 ETA trainers (eAppendix 2 in Supplement 2).

Three of us (S.B.B., E.L.M., and A.M.S.) independently developed a list of codes describing recurring ideas in the focus group transcripts and then agreed on a consensus code list for the teacher (20 codes) and the trainer (15 codes) focus groups. A code was designated as a theme (11 for teachers and 10 for trainers) if more than half of the focus group participants made at least 1 comment to which that code was applied. Two of us (R.C.W. and A.N.H.) identified teacher themes that supported 1 or more outcome constructs in the conceptual framework (eFigure in Supplement 2) and any teacher or trainer themes that supported implementation fidelity for the ETA curriculum.

## Results

Overall, 96 teachers (93 [96.9%] women; 58 [60.4%] aged ≥ 40 years) from 63 classrooms (38 sites) agreed to participate (Figure). The neighborhood characteristics for the 38 sites with participating teachers were similar to those of the 95 eligible sites without participating teachers (eTable 2 in Supplement 2). During randomization, 32 classrooms (48 teachers) were allocated to the intervention group and 31 classrooms (48 teachers) to the control group. Both lead and assistant teachers participated in 33 of 63 classrooms (52.4%). There was 1 participating teacher in 16 intervention classrooms (6 with lead teacher only and 10 with assistant teacher only) and in 14 control classrooms (6 with lead teacher only and 8 with assistant teacher only). The follow-up survey was completed by 47 teachers (97.9%) in the intervention group and 46 teachers (95.8%) in the control group. Overall, 93 of 96 teachers (96.9%; 61 of 63 classrooms [96.8%]) completed the survey (Figure). The 3 teachers with missing follow-up data (2 control teachers and 1 intervention teacher) were excluded from the impact analyses. Of 7 teachers (4 lead and 3 assistant) in the intervention group who attended no ETA sessions, 6 completed the follow-up survey, and their outcome data were analyzed along with others in the intervention group.

**Figure.**
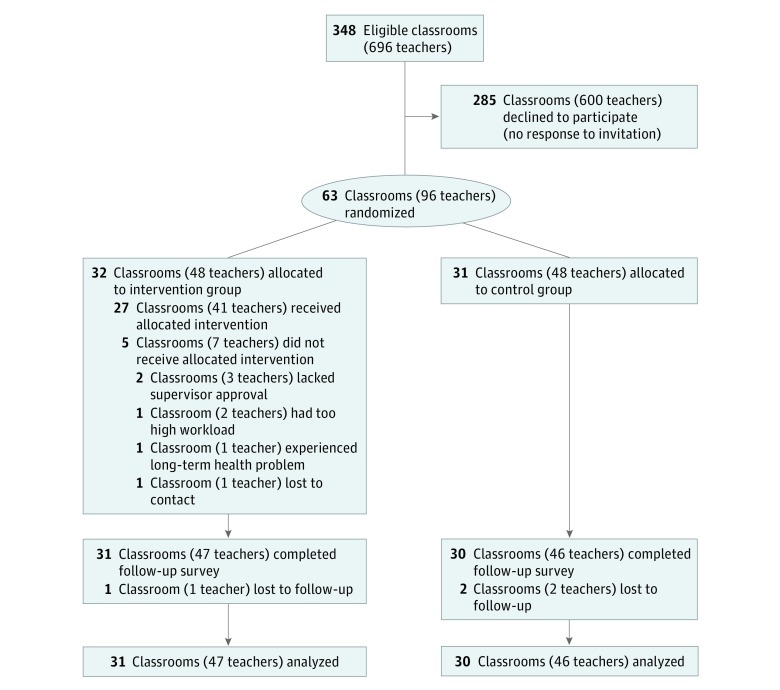
CONSORT Flowchart Describing Progress of Participants Through the Study

Of 96 teachers enrolled, 48 (49.9%) had at least a bachelor’s degree (Table 1); 35 (36.5%) reported having 3 or more categories of ACEs; 51 (53.1%) had children of their own who had attended Head Start, and 31 (33.0%) reported not having enough money to pay their full utility bill sometime in the prior 12 months. Of 48 teachers assigned to the intervention, 38 (79.2%) attended 4 or more of the 6 ETA sessions and 7 (14.6%) attended no sessions. No harms were reported.

**Table 1.** Baseline Characteristics of Study Participants

Characteristic	No. (%)^a^^,^^b^		
	Total (N = 96)	Intervention Group (n = 48)	Control Group (n = 48)
Women	93 (96.9)	46 (95.8)	47 (97.9)
Age group, y			
≤29	19 (20.0)	5 (10.6)	14 (29.2)
30-39	18 (18.9)	13 (27.7)	5 (10.4)
40-49	19 (20.0)	8 (17.0)	11 (22.9)
50-59	30 (31.6)	16 (34.0)	14 (29.2)
≥60	9 (9.5)	5 (10.6)	4 (8.3)
Race/ethnicity			
White, non-Hispanic	24 (25.3)	10 (21.3)	14 (29.2)
Black, non-Hispanic	54 (56.8)	31 (66.0)	23 (47.9)
Other race, non-Hispanic^c^	2 (2.1)	1 (2.1)	1 (2.1)
Hispanic, any race	15 (15.8)	15 (10.6)	10 (20.8)
Highest level of education			
High school or GED degree	22 (22.9)	12 (25.0)	10 (20.8)
Associate’s degree	26 (27.1)	13 (27.1)	13 (27.1)
Bachelor’s degree	30 (31.2)	13 (27.1)	17 (35.4)
Master’s or doctoral degree	18 (18.7)	10 (20.8)	8 (16.7)
ECE work experience, y			
0-6	24 (25.5)	10 (21.7)	14 (29.2)
7-15	25 (26.6)	13 (28.3)	12 (25.0)
>15	45 (47.9)	23 (50.0)	22 (45.8)
Lead teacher	45 (46.9)	22 (45.8)	23 (47.9)
Own child attended Head Start	51 (53.1)	25 (52.1)	26 (54.2)
Works another job for pay	15 (15.6)	5 (10.4)	10 (20.8)
Received SNAP	19 (20.0)	9 (19.1)	10 (20.8)
Not enough money for housing	15 (16.0)	3 (6.5)	12 (25.0)
Not enough money for utilities	31 (33.0)	15 (32.6)	16 (33.3)
Not enough money for health care	22 (23.4)	7 (15.2)	15 (31.2)
Categories of ACEs, No.^d^			
0	20 (20.8)	9 (18.7)	11 (22.9)
1	14 (14.6)	9 (18.7)	5 (10.4)
2	27 (28.1)	11 (22.9)	16 (33.3)
3	9 (9.4)	5 (10.4)	4 (8.3)
≥4	26 (27.1)	14 (29.2)	12 (25.0)
Teaches in a Head Start classroom^e^	64 (66.7)	26 (54.2)	38 (79.2)

Abbreviations: ACEs, adverse childhood experiences; ECE, early childhood education; GED, passed General Education Development Test; SNAP, Supplemental Nutrition Assistance Program.

^a^ Participants were missing data on characteristics as follows: age (n = 1), race/ethnicity (n = 1), work experience (n = 2), received SNAP (n = 1), not enough money for housing (n = 2), not enough money for utilities (n = 2), not enough money for health care (n = 2).

^b^ Percentages across levels of a characteristic may not add to 100.0 owing to rounding.

^c^ Includes participants who reported not being of Hispanic origin and reported 1 of the following: American Indian or Alaska Native; Asian; Native Hawaiian or other Pacific Islander; biracial or multiracial; or other.

^d^ Count is based on the experience of up to 10 categories of ACEs before age 18 years: emotional abuse, physical abuse, sexual abuse, household mental illness, incarcerated household member, household substance abuse, parental separation or divorce, intimate partner violence, emotional neglect, and physical neglect. For 8 categories, items were worded and scored in accordance with the Centers for Disease Control and Prevention’s Behavioral Risk Factor Surveillance Survey.^31,32^ For the categories of emotional and physical neglect, items were developed using the wording and scoring described elsewhere.^33^

^e^ Overall, 43 of 63 classrooms (68.2%) were Head Start classrooms (18 of 32 [56.2%] intervention classrooms and 25 of 31 [80.6%] control).

### Primary Outcome Evaluation

Adjusting for baseline values, conflict scores following the intervention did not differ significantly between the intervention and control groups (Table 2). The adjusted mean (SE) conflict scores tended to be higher for those randomly assigned to the intervention than for controls (15.8 [0.6] vs 15.0 [0.6]; effect size = 0.16; 95% CI, −0.19 to 0.52). There were no statistically significant effects of the intervention on relational trust with other adults (3 outcomes), nor were there significant effects in the domains of relational capacity (8 outcomes) or health and well-being (10 outcomes) (Table 2).

**Table 2.** Effect of Enhancing Trauma Awareness Course on Measures of Relationship Quality, Relational Capacities, and Health and Well-being

Variable and Hypothesized Direction of Change^a^	Baseline, Mean (SD)		Follow-up, Mean (SD)		Mean or Prevalence Difference at Follow-up (Intervention vs Control)			Magnitude of Effect (95% CI)^d^
	Intervention (n = 47)	Control (n = 46)	Intervention (n = 47)	Control (n = 46)	Unadjusted (95% CI)	Adjusted (95% CI)^b^	*P* Value^c^	
**Relationship Quality**								
Decreased teacher-children conflict	15.8 (5.1)	15.9 (5.9)	15.7 (5.2)	15.2 (3.7)	0.5 (−1.3 to 2.4)	0.7 (−0.8 to 2.3)	.36	0.16 (−0.19 to 0.52)
Trust with adults								
Increased trust with parents	10.3 (1.4)	10.2 (1.2)	10.2 (1.4)	9.9 (1.4)	0.3 (−0.3 to 0.9)	0.2 (−0.3 to 0.7)	.38	0.16 (−0.19 to 0.51)
Increased trust with other staff	5.4 (0.9)	5.3 (0.9)	5.4 (0.9)	5.3 (0.9)	0.1 (−0.3 to 0.4)	0 (−0.3 to 0.3)	.97	0.01 (−0.32 to 0.33)
Increased trust with supervisors	7.2 (1.3)	7.1 (1.4)	6.9 (1.5)	7.0 (1.5)	−0.1 (−0.7 to 0.4)	−0.2 (−0.6 to 0.2)	.27	−0.17 (−0.47 to 0.13)
**Relational Capacities**								
Emotion regulation								
Increased cognitive reappraisal	5.3 (1.2)	5.1 (1.0)	5.1 (1.1)	5.0 (1.1)	0.1 (−0.3 to 0.5)	−0.02 (−0.4 to 0.4)	.91	−0.02 (−0.39 to 0.35)
Decreased expressive suppression	3.3 (1.1)	3.4 (1.3)	3.2 (1.2)	3.4 (1.1)	−0.2 (−0.7 to 0.3)	−0.2 (−0.6 to 0.3)	.44	−0.14 (−0.51 to 0.22)
Increased dispositional mindfulness	38.0 (5.8)	37.0 (5.8)	36.2 (5.6)	36.4 (5.6)	−0.2 (−2.5 to 2.2)	−1.1 (−2.9 to 0.6)	.20	−0.21 (−0.52 to 0.11)
Empathy								
Increased perspective-taking	21.3 (3.8)	20.7 (4.0)	20.1 (4.0)	20.3 (4.3)	−0.1 (−1.9 to 1.6)	−0.7 (−2.2 to 0.8)	.35	−0.17 (−0.54 to 0.19)
Increased empathic concern	23.3 (3.0)	22.0 (3.7)	22.7 (4.2)	22.5 (3.9)	0.2 (−1.5 to 1.9)	−0.6 (−2.0 to 0.9)	.44	−0.14 (−0.50 to 0.22)
Decreased personal distress	8.8 (4.7)	8.8 (5.2)	9.4 (5.3)	9.1 (4.6)	0.3 (−1.8 to 2.3)	0.3 (−1.3 to 1.8)	.73	0.05 (−0.26 to 0.37)
Increased compassion satisfaction	49.0 (11.0)	50.9 (9.2)	49.0 (9.7)	51.0 (10.3)	−1.9 (−6.1 to 2.2)	−0.9 (−4.0 to 2.2)	.56	−0.09 (−0.40 to 0.22)
Increased ARTIC	5.6 (1.0)	5.5 (0.9)	5.5 (1.2)	5.2 (0.9)	0.3 (−0.1 to 0.8)	0.3 (−0.04 to 0.64)	.08	0.29 (−0.04 to 0.61)
**Health and Well-being**								
Burnout								
Decreased emotional exhaustion	1.8 (1.2)	1.9 (1.4)	1.7 (1.2)	1.8 (1.3)	−0.04 (−0.55 to 0.48)	−0.02 (−0.38 to 0.35)	.93	−0.01 (−0.31 to 0.28)
Decreased depersonalization	0.5 (0.7)	0.7 (1.0)	0.6 (0.8)	0.7 (0.9)	−0.2 (−0.5 to 0.2)	−0.05 (−0.38 to 0.28)	.77	−0.06 (−0.44 to 0.33)
Increased personal accomplishment	5.2 (0.7)	5.0 (0.8)	5.0 (0.9)	5.1 (0.9)	−0.1 (−0.5 to 0.3)	−0.2 (−0.5 to 0.1)	.20	−0.22 (−0.56 to 0.12)
Decreased secondary traumatic stress	51.2 (10.0)	48.8 (10.4)	52.1 (10.7)	47.9 (8.8)	4.2 (0.1 to 8.3)	3.2 (−0.2 to 6.7)	.07	0.32 (−0.02 to 0.67)
Increased job satisfaction	5.2 (1.1)	5.5 (0.6)	4.7 (1.4)	5.1 (1.1)	−0.4 (−0.9 to 0.1)	−0.4 (−1.0 to 0.1)	.13	−0.34 (−0.77 to 0.09)
Health-related quality of life, No. (%)								
Decrease in any mentally unhealthy days	28 (60.9)	24 (52.2)	30 (65.2)	31 (67.4)	−2.2 (−21.5 to 17.1)	−5.7 (−24.1 to 12.7)	.55	0.73 (0.26 to 2.04)
Decrease in any physically unhealthy days	22 (46.8)	23 (50.0)	30 (63.8)	32 (69.6)	−5.7 (−24.8 to 13.4)	−4.4 (−29.3 to 20.5)	.73	0.75 (0.14 to 3.88)
Decrease in any days poor health interferes	11 (23.4)	11 (24.4)	23 (48.9)	21 (46.7)	2.3 (−18.1 to 22.7)	4.8 (−24.6 to 34.2)	.75	1.24 (0.34 to 4.56)
Sleep								
Increase in sleep duration to ≥7 h/night, No. (%)	21 (45.6)	17 (37.8)	16 (34.8)	19 (42.2)	−7.4 (−27.4 to 12.5)	−10.8 (−28.7 to 7.0)	.24	0.56 (0.21 to 1.48)
Increased quality	2.9 (0.7)	2.8 (0.6)	2.8 (0.6)	2.8 (0.6)	0.1 (−0.2 to 0.3)	0.03 (−0.18 to 0.24)	.78	0.05 (−0.30 to 0.40)

Abbreviation: ARTIC, attitudes related to trauma-informed care.

^a^ For teacher-children conflict, mindfulness, job satisfaction, and physically unhealthy days measures, n = 93 (no missing values). For trust with parents and staff, emotion regulation, empathy, compassion satisfaction, burnout, secondary traumatic stress, mentally unhealthy days, and days poor health interferes measures, n = 92. For trust with supervisors, ARTIC, and sleep measures, n = 91.

^b^ Adjusted mean differences for continuous outcomes control for baseline level of the outcome variable and account for clustering of teacher outcomes at classroom and site levels. Adjusted prevalence differences for binary outcomes (mentally unhealthy days, physically unhealthy days, days poor health interferes, and sleep duration) were determined from predictive probabilities using only the fixed portion of the model.

^c^ The *P* value is associated with the regression coefficient for the study group variable (intervention[1]/control[0]) in each linear multilevel (classroom and site levels) regression model (continuous outcomes) and with the odds ratio in each logistic multilevel (classroom and site levels) regression model (binary outcomes). A significance threshold of *P* < .05 from 2-sided testing was used.

^d^ The magnitude of the effect refers to an effect size. Effect-size measures (adjusted for baseline values and clustering) were derived in linear models from the standardized partial coefficient for the binary study group variable and in logistic models from the odds ratio for that binary variable (mentally unhealthy days, physically unhealthy days, days poor health interferes, and sleep duration).

### Secondary Analysis

This study was not designed to examine the effect of the intervention on subgroups, so subgroup analyses were not specified before the trial and were exploratory. The effect of the intervention on conflict scores did not differ significantly between teacher subgroups defined on the following binary (yes/no) characteristics (eTable 3 in Supplement 2): worked in a site with both intervention and control classrooms (yes: adjusted mean difference [MD], 0.5; 95% CI, −2.1 to 3.2; no: MD, 0.6; 95% CI, −1.4 to 2.6; *P* = .89); lead teacher (yes: MD, −0.05; 95% CI, −2.1 to 2.0; no: MD, 1.1; 95% CI, −0.9 to 3.1; *P* = .43); more than 15 years of early childhood teaching experience (yes: MD, 0.5; 95% CI, −1.7 to 2.8; no: MD, 0.6; 95% CI, −1.5 to 2.8; *P* = .95); 3 or more ACEs (yes: MD, 1.0; 95% CI, −1.0 to 3.0; no: MD, 0.3; 95% CI, −1.8 to 2.3; *P* = .61); and worked in a Head Start classroom (yes: MD, 1.8; 95% CI, −0.3 to 3.8; no: MD, −0.6; 95% CI, −3.0 to 1.8; *P* = .16). However, the effect of the intervention on conflict scores differed significantly by teacher education level. After exposure to ETA, teachers with less than a bachelor’s degree had significantly more conflict (MD, 2.5; 95% CI, 0.4 to 4.6), while those with more education tended to have less conflict (MD, −1.3; 95% CI, −3.2 to 0.5) after taking the course (*P* = .01).

At delayed follow-up, there were no statistically significant effects of the intervention on conflict scores or any secondary outcomes (eTable 4 in Supplement 2). The changes between baseline and delayed follow-up were similar between those in the intervention group who participated in the focus groups and those who did not (eTable 5 in Supplement 2).

### Exploratory Focus Group Evaluation

The outcomes in our conceptual model (eFigure in Supplement 2) were supported by 7 of 11 teacher themes in the focus groups (Table 3; eTable 6 in Supplement 2). The teachers’ comments in the focus groups suggested that ETA helped with the quality of their relationships with children and parents and that after taking the course teachers had greater empathy, emotion regulation, and mindfulness as well as improved attitudes related to trauma-informed care. Describing the course as a healing process, teachers reported that they became aware of the effects of trauma in their own lives and thereby developed greater empathy for children and parents. After taking the course, teachers viewed more of children’s experiences as being potentially traumatic and more often recognized the potential effect of trauma in their nonwork relationships. Teachers reported that they acquired skills in responding compassionately to challenging situations at school that may have arisen from trauma outside of school. Teachers reported on their new abilities to step back, maintain a calm presence in the moment, and avoid interpreting the challenging behaviors of children or parents as reflecting poorly on teacher performance. In addition, teachers mentioned taking care of themselves as a way to better regulate their emotions and, in turn, to create emotionally safe classrooms characterized by less conflict and more nurturance and support.

**Table 3.** Qualitative Themes Mapping Onto the Conceptual Framework

Model Construct	Qualitative Theme (No. of Speakers)^a^	Example Quote
**Relationship Quality**		
Teacher-children conflict	Theme 9: More mindful of trauma in managing the classroom(14)	Participant 2, FG 2, themes 9 and 11: “I think what I took away from it [the ETA course] is to be a little more laid back and understanding. I may have something planned at a certain time, but like, taking the tone and temperature of the room and the moods, that might not be the best time, and when people come in, it’s not all about you. Sometimes it’s about them and if I haven’t done anything to you and I haven’t seen you that morning and you’re angry, evidently there’s some other things going on, and not take it so personally. People have things going on. And not let that affect me. How I speak to them or whatever, just more of a laid back approach, it’s not all about me, other people are going through things. And you don’t know what it might be. They may share they may not. But just to have that understanding that, it’s okay, sometimes you can take that extra minute, if it’s not done at 9:25, it can be done at 9:30, just that type of thing, and just a little more laid back. If it makes it calmer and more settled and more comforting, it’s helpful, and I don’t feel guilty about it.”
	Theme 11: Created a more emotionally safe classroom (13)	
Trust with parents	Theme 8: More sensitive to behaviors reflecting responses to trauma (15)	Participant 3, FG 2, theme 8: “…and I have some parents that don’t like me very much. I don’t know why, but they’re getting more upset than I am. And I’m like, it’s okay, I’m not upset. Because what I’m looking at, is what you were talking about, that iceberg [an analogy presented in the ETA course to suggest that emotions arising from past experiences may be occurring below the surface of an individual’s outward behavior], there’s something going on with their parent, so I’m not gonna judge them. If they don’t like me, it’s okay, but I’m still gonna treat them how I want them to treat me…. Sooner or later I’m gonna break down that barrier, which I have done, and I’m gonna be okay, so I just let it happen, and I’m not bothered by it…. The other teachers are really getting upset. I’m like ‘it’s okay.’ No, I’m not gonna be mad, because I know something is going on, and I think that, having this training really helped a lot. It really did.”
**Relational Capacities**		
Empathy	Theme 5:Greater understanding of children’s experiences as traumatic (10)	Participant 8, FG 1, themes 5, 8, and 9: “And you don’t know if 5 kids are sleeping in 1 bed, and the class [ETA course] taught you to just back off and listen and see where the child was coming from. And then try to deal with them where they were, as far [as] I guess, mentally, or whatever. I’m a sensitive person, but it [the ETA course] just made you step back and be more caring, even if you’re caring, more caring. Yeah, to their needs.” Participant 7, FG 2, themes 7, 8, and 9: “How it has changed for me is, you always hear the statement ‘you never know how someone is until you walk a mile in their shoes’ and I feel like there is a lot of truth to that because if you can learn how to retrain your mind to think of how somebody else might be feeling, it helps you maybe not to approach that person in a negative way. We all learned to step back and just imagine to a degree, okay, I don’t know what they’re going through, and it enables you to try different ways to approach that person. So at home or at work I’m constantly trying to see things from other people’s perspectives. And I’m trying to not assume anything because everybody has a different type of trauma.… I do the same thing at home. I’m trying to help my husband understand that kind of stuff too because he’s a person that likes to just jump right in and just assume negative things…. You gotta learn to think that just because you see the surface, that there aren’t other things that are going on underneath of it, just like there’s stuff going on with you. So I’m trying to take those strategies and use them everywhere I go, because I really feel like it’s really beneficial, so this was definitely a helpful program that I think should be mandatory…. The world is full of trauma. Full of trauma.”
	Theme 7: Greater recognition of trauma in nonwork relationships (10)	
	Theme 8: More sensitive to behaviors reflecting responses to trauma (15)	
	Theme 9: More mindful of trauma in managing the classroom (14)	
Emotion regulation	Theme 3: Experienced healing from the course (9)	Participant 1, FG 2, themes 9 and 10: “Taking a step back and taking a break, taking deep breaths and thinking about the problem instead of just rushing to solve the problem. You can’t solve the problem like that. If you are upset, take a step back and relax first, but how to relax, well I didn’t really know a lot of techniques. I would go home with lots of baggage, lots of heaviness, lots of problems. I would take all the children’s problems, all the parents’ problems at home…. I went home with almost every day, not every day, but [with] headaches. I do get headaches a lot when I get tension, when I get stressed out. I’ve been really aware of my body now which I was not before, and I know when I do get upset and I kind of step back and I see it differently and I tell [my coteacher] I need 1 minute. I go in the office, sip a little bit coffee and then come out, but it makes a big difference. And I love that [my coteacher] and I, we work together because… we talk to each other, and we calm each other down. So this is another plus, that the course has really taught us how to really be aware of the whole environment not only in the class, at home too. I mean with our husbands or our wives or whoever. You have to take a step back and take some deep breaths before you rush.” Participant 6, FG 1, themes 3, 8, and 9: “The tissue box was passed around [during ETA sessions] in a positive way because it brought things out that you had inside you that you didn’t realize that you had, and you should’ve gotten it out, and it makes you more aware and sensitive to others’ needs…. You get these nasty mouth ones or the wild ones or some of these special needs [children] that are off the hook, it teaches you how to not attack them, but to calm them, and when they’re at high trauma, you want to keep it down, so it won’t affect their minds or affect your mind. Even though I’m more of a calm person anyways, it even brought me down some more.”
	Theme 9: More mindful of trauma in managing the classroom (14)	
	Theme 10: Used self-care strategies to remain calm (9)	
Dispositional mindfulness	Theme 9: More mindful of trauma in managing the classroom (14)	Participant 6, FG 2, themes 8 and 9: “I think before the [ETA] course, I was more, first to react to a situation and then think. But after the course I sat back and then I thought and I approached the situation. I think that’s something that I did differently, knowing how trauma is, and what it looks like and me not knowing [that] before, I would always react to a situation not knowing what the trauma was, and not understanding that I might have been, also, exacerbating the situation.”
Attitudes about trauma-informed care	Theme 5: Greater understanding of children’s experiences as traumatic (10)	Participant 3, FG 1, themes 8 and 9: “…and from the time that we were raised and how we were taught certain things when adults talk to you, you just did. Now we have [children with] attitudes and stuff when they are just not gonna do it, and initially we just think they are being grown and being defiant, but now you know, okay there’s a reason why these children are acting this way. So that’s the difference for me it made, to look at them differently, their behaviors differently, what is making you act this way, and yes, and back to the triggers, you know. I have one [student], there’s a way to talk to him and handle things with him because he could go loose, real fast, you know what I’m talking about. You have screaming and howling over here, kicking, but if you calmly talk to him and rub his back a little bit, most of the time it helps. Sometimes it doesn’t, but we have some good days now, instead of a lot of days, you know, being rough.”
	Theme 8: More sensitive to behaviors reflecting responses to trauma (15)	

Abbreviations: ETA, Enhancing Trauma Awareness; FG, focus group.

^a^ Indicates the number of focus group participants (of 15) who had at least 1 comment during the focus group that supported the theme.

Overall, 6 of 11 teacher themes and 6 of 10 trainer themes supported the implementation fidelity of ETA (eAppendix 3 in Supplement 2). Teachers reported that the course content helped them reconceptualize trauma, increasing their awareness that teachers, children, and families had all experienced trauma. Teachers’ awareness of their own trauma was facilitated in the course by what both teachers and trainers described as a supportive emotional climate characterized by nurturance and respect. This climate allowed teachers to safely share and apply the information learned in the course. The cotrainers modeled for the teachers the relational capacities necessary for teachers to transform their own relationships and express more compassion toward others and themselves.

## Discussion

In a randomized clinical trial, we evaluated a 6-session professional development course to increase trauma-informed care among preschool teachers working with low-income children and families. We found no significant effects of the course on reducing teacher-children conflict scores or on the secondary outcomes assessing teachers’ relational capacities or their health and well-being.

The focus group evaluation was exploratory, involved only 15 of 41 teachers (36.6%) who received the intervention, and was not intended to have a control or comparison group. With these limitations in mind, the themes from the focus groups suggested that after taking the course the teachers believed that they experienced improvements in the quality of their relationships with children. Teachers described a healing process of becoming aware of the effect of trauma in their own lives and thereby developing greater empathy for children and parents. Teachers reported that these shifts in their perspectives led them to create more emotionally safe classrooms by remaining calmer and more mindful in the face of children’s challenging behaviors that may have reflected trauma.

In a systematic review of trauma-informed organizational changes that included staff trainings, only 4 randomized clinical trials were identified, and none were in educational settings.^4^ In 2 trials with inpatient psychiatric staff, no impact of staff training on staff trauma sensitivity^35^ or knowledge about traumatic stress^36^ was found. In a third trial with 148 outpatient staff at a clinic treating substance use disorders,^37^ there was an increase in staff knowledge of trauma and confidence in asking about trauma, but there was a low follow-up rate in both study groups. Finally, in a trial with 30 primary care physicians, there were positive impacts on patient-physician relationship quality, as measured by patients’ perceptions of more shared decision making with their physicians^38^ and more patient centeredness on direct observation.^39^ The only reviewed study that was in an educational setting^40^ involved multiple trauma-informed interventions (including staff trainings) in a single elementary school without a control group or staff assessments.

We can only speculate about why we obtained different results from the survey and focus group data. Compared with reporting in an online survey, teachers may have been more likely to report positively about the intervention during focus groups. Focus group participants may have been a biased sample among those assigned to the intervention, and the focus groups were conducted 5 months after the intervention. However, we found similar survey-based outcomes in focus group and non–focus group participants 5 months after the intervention. In interventions that aim to increase trauma-informed care, some meaningful and enduring effects may not be captured by surveys. The change a person experiences as a result of an intervention might only be captured through a narrative about how their specific relationships change over time. Furthermore, this expression of change might only be possible in a group in which emotional safety has already been established and/or in which the story of change emerges in a dialogue with others who have experienced similar changes.

### Limitations

The trial had design limitations. There was no masking of participants, no active control group, and all survey outcomes were self-reported. We may have obtained different results if the teachers had completed the conflict scale for individual students^29^ rather than for the class as an aggregate.^30^ We recruited fewer teachers for the trial than planned, but the observed effect size of the primary outcome was still much smaller than the planned minimal detectable effect size. Approximately one-third of the participants were not from a classroom where both teachers participated, so some of the teacher data were not clustered at the classroom level. The control group was offered ETA in the same year as the intervention group, so there was no untreated comparison group in the delayed follow-up survey. We did not conduct classroom observations. However, in the exploratory focus groups, the teachers described a shift in their internal experience of relationships, and the behavioral manifestations of such changes may have been difficult to observe. Finally, interventions to institute trauma-informed practices may have greater effects if they involve the entire organization, including supervisors, and change the way individuals in organizations relate to each other.^17^

## Conclusions

In this trial, a professional development course to enhance trauma awareness among preschool teachers did not change their perceptions of conflict with children in their classrooms. Future research on professional development courses to increase trauma-informed care may benefit from using both quantitative and qualitative assessments, including data collected from those receiving services. Although changes in attitudes may be a legitimate goal for professional development courses, changes in behavior and relationship quality may require more sustained training, involving skill acquisition through practice and coaching. Approaches are also needed to sensitively identify when or if professionals are ready to participate in courses like ETA or how any individual will respond to a course that may make them become aware of their own traumatic experiences. Therefore, priority must be given to the training of those delivering such courses, so that the courses combine trauma knowledge with a relational process that provides safety for participants.

## Notes

Supplement 1. **Trial Protocol**

Supplement 2. **eFigure. **Conceptual Framework Guiding the Evaluation of Enhancing Trauma Awareness**** **eAppendix 1. **Description of the Enhancing Trauma Awareness Course **eAppendix 2. **Focus Group Methods for Evaluating the Enhancing Trauma Awareness Course **eAppendix 3. **Qualitative Themes Supporting Fidelity to the Enhancing Trauma Awareness Course Content and Relational Process **eTable 1. **Detailed Instrument Descriptions for the Trial Outcome Measures **eTable 2. **Neighborhood Characteristics of Eligible Early Childhood Education Sites, by Participation in the Trial **eTable 3. **Subgroup Comparisons of the Effect of Enhancing Trauma Awareness on Teacher-Children Conflict Scores**** **eTable 4. **Effect of Enhancing Trauma Awareness at Delayed Follow-up on Measures of Relationship Quality, Relational Capacities, and Health and Well-being **eTable 5. **Changes in Outcome Measures Between Baseline and Delayed Follow-up for Those in the Intervention Group Who Did and Did Not Participate in Focus Groups **eTable 6. **Additional Teacher Focus Group Quotes Supporting Outcomes in the Conceptual Framework **eReferences**

Supplement 3. **Data Sharing Statement**

## References

[1] Substance Abuse and Mental Health Services Administration (SAMHSA) SAMHSA’s concept of trauma and guidance for a trauma-informed approach. https://store.samhsa.gov/system/files/sma14-4884.pdf. Accessed January 16, 2019.

[2] Anda RF, Felitti VJ, Bremner JD, The enduring effects of abuse and related adverse experiences in childhood: a convergence of evidence from neurobiology and epidemiology. Eur Arch Psychiatry Clin Neurosci. 2006;256(3):-. doi:10.1007/s00406-005-0624-4 16311898 PMC3232061

[3] Bethell CD, Solloway MR, Guinosso S, Prioritizing possibilities for child and family health: an agenda to address adverse childhood experiences and foster the social and emotional roots of well-being in pediatrics. Acad Pediatr. 2017;17(7S):S36-S50. doi:10.1016/j.acap.2017.06.002 28865659

[4] Purtle J Systematic review of evaluations of trauma-informed organizational interventions that include staff trainings. Trauma Violence Abuse. 2018;1524838018791304. doi:10.1177/1524838018791304 30079827

[5] Hughes K, Bellis MA, Hardcastle KA, The effect of multiple adverse childhood experiences on health: a systematic review and meta-analysis. Lancet Public Health. 2017;2(8):e356-e366. doi:10.1016/S2468-2667(17)30118-4 29253477

[6] Felitti VJ, Anda RF, Nordenberg D, Relationship of childhood abuse and household dysfunction to many of the leading causes of death in adults: the Adverse Childhood Experiences (ACE) study. Am J Prev Med. 1998;14(4):245-258. doi:10.1016/S0749-3797(98)00017-8 9635069

[7] Shonkoff JP, Boyce WT, McEwen BS Neuroscience, molecular biology, and the childhood roots of health disparities: building a new framework for health promotion and disease prevention. JAMA. 2009;301(21):2252-2259. doi:10.1001/jama.2009.754 19491187

[8] Danese A, McEwen BS Adverse childhood experiences, allostasis, allostatic load, and age-related disease. Physiol Behav. 2012;106(1):29-39. doi:10.1016/j.physbeh.2011.08.019 21888923

[9] Fang X, Brown DS, Florence CS, Mercy JA The economic burden of child maltreatment in the United States and implications for prevention. Child Abuse Negl. 2012;36(2):156-165. doi:10.1016/j.chiabu.2011.10.006 22300910 PMC3776454

[10] Lê-Scherban F, Wang X, Boyle-Steed KH, Pachter LM Intergenerational associations of parent adverse childhood experiences and child health outcomes. Pediatrics. 2018;141(6):1-9. doi:10.1542/peds.2017-4274 29784755

[11] Randell KA, O’Malley D, Dowd MD Association of parental adverse childhood experiences and current child adversity. JAMA Pediatr. 2015;169(8):786-787. doi:10.1001/jamapediatrics.2015.0269 26030177

[12] Halfon N, Larson K, Son J, Lu M, Bethell C Income inequality and the differential effect of adverse childhood experiences in US children. Acad Pediatr. 2017;17(7S):S70-S78. doi:10.1016/j.acap.2016.11.007 28865663

[13] Shonkoff JP, Garner AS; Committee on Psychosocial Aspects of Child and Family Health; Committee on Early Childhood, Adoption, and Dependent Care; Section on Developmental and Behavioral Pediatrics The lifelong effects of early childhood adversity and toxic stress. Pediatrics. 2012;129(1):e232-e246. doi:10.1542/peds.2011-2663 22201156

[14] National Center for Injury Prevention and Control, Division of Violence Prevention, Centers for Disease Control and Prevention Essentials for childhood: steps to create safe, stable, nurturing relationships and environments. https://www.cdc.gov/violenceprevention/pdf/essentials_for_childhood_framework.pdf. Accessed January 16, 2019.

[15] Valent P Survival strategies: a framework for understanding secondary traumatic stress and coping in helpers In: Figley CR, ed. Compassion Fatigue: Coping with Secondary Traumatic Stress Disorder in Those Who Treat the Traumatized. New York, NY: Routledge; 2015:21-50.

[16] Geller SM, Porges SW Therapeutic presence: neurophysiological mechanisms mediating feeling safe in therapeutic relationships. J Psychother Integration. 2014;24(3):178-192. doi:10.1037/a0037511

[17] Bloom SL Creating Sanctuary: Toward the Evolution of Sane Societies. Revised ed. New York, NY: Routledge; 2013.

[18] Levenson J Trauma-informed social work practice. Soc Work. 2017;62(2):105-113. doi:10.1093/sw/swx001 28339563

[19] Mashburn AJ, Hamre BK, Downer JT, Pianta RC Teacher and classroom characteristics associated with teachers’ ratings of prekindergartners’ relationships and behaviors. J Psychoed Assess. 2006;24(4):367-380. doi:10.1177/0734282906290594

[20] Van der Kolk BA Developmental trauma disorder: toward a rational diagnosis for children with complex trauma histories. Psychiatr Ann. 2005;35(5):401-408. doi:10.3928/00485713-20050501-06

[21] Herman JL Trauma and Recovery: The Aftermath of Violence: From Domestic Abuse to Political Terror. New York, NY: Basic Books; 2015.

[22] Bath H The three pillars of trauma-informed care. Reclaiming Child Youth. 2008;17(3):17-21. https://s3-us-west-2.amazonaws.com/cxl/backup/prod/cxl/gklugiewicz/media/507188fa-30b7-8fd4-aa5f-ca6bb629a442.pdf. Accessed March 20, 2019.

[23] Perry BD Applying principles of neurodevelopment to clinical work with maltreated and traumatized children: the neurosequential model of therapeutics In: Webb NB, ed. Working With Traumatized Youth in Child Welfare. New York, NY: Guilford Press; 2006:27-52.

[24] Rosenbloom D, Williams MB, Watkins BE Life After Trauma: A Workbook for Healing. 2nd ed New York, NY: Guilford Press; 2010.

[25] Arvidson J, Kinniburgh K, Howard K, Treatment of complex trauma in young children: developmental and cultural considerations in application of the ARC intervention model. J Child Adolesc Trauma. 2011;4(1):34-51. doi:10.1080/19361521.2011.545046

[26] Napier R, Gershenfeld MK Groups: Theory and Experience. Boston, MA: Houghton Mifflin; 2004.

[27] Silberman ML, Biech E, Auerbach C Active Training: A Handbook of Techniques, Designs, Case Examples and Tips. 4th ed Hoboken, NJ: John Wiley & Sons, Inc; 2015. doi:10.1002/9781119154778

[28] Knowles MS, Holton EF, Swanson RA The Adult Learner: The Definitive Classic in Adult Education and Human Resource Development. New York, NY: Routledge; 2012. doi:10.4324/9780080964249

[29] Pianta RC Student-Teacher Relationships Scale: Professional Manual. Lutz, FL: Psychological Assessment Resources; 2001.

[30] Whitaker RC, Dearth-Wesley T, Gooze RA Workplace stress and the quality of teacher–children relationships in Head Start. Early Child Res Q. 2015;30(pt A):57-69. doi:10.1016/j.ecresq.2014.08.008

[31] US Centers for Disease Control and Prevention Violence prevention: about Behavioral Risk Factor Surveillance System ACE data. https://www.cdc.gov/violenceprevention/acestudy/ace_brfss.html. Accessed January 16, 2019.

[32] Bynum L, Griffin T, Riding D, ; Centers for Disease Control and Prevention Adverse childhood experiences reported by adults: five states, 2009. MMWR Morb Mortal Wkly Rep. 2010;59(49):1609-1613. 21160456

[33] Stevens JE Got your ACE score? https://acestoohigh.com/got-your-ace-score/. Accessed January 16, 2019.

[34] Lorah J Effect size measures for multilevel models: definition, interpretation, and TIMSS example. Large Scale Assess Educ. 2018;6(1):1. doi:10.1186/s40536-018-0061-2

[35] Borckardt JJ, Madan A, Grubaugh AL, Systematic investigation of initiatives to reduce seclusion and restraint in a state psychiatric hospital. Psychiatr Serv. 2011;62(5):477-483. doi:10.1176/ps.62.5.pss6205_0477 21532072

[36] Crable AR, Underwood LA, Parks-Savage A, Maclin V An examination of a gender-specific and trauma-informed training curriculum: implications for providers. Int J Behav Consult Ther. 2013;7(4):30-37. doi:10.1037/h0100964

[37] Lotzin A, Buth S, Sehner S, ; CANSAS Study Group “Learning how to ask”: effectiveness of a training for trauma inquiry and response in substance use disorder healthcare professionals. Psychol Trauma. 2018;10(2):229-238. doi:10.1037/tra0000269 28581317

[38] Green BL, Saunders PA, Power E, Trauma-informed medical care: patient response to a primary care provider communication training. J Loss Trauma. 2016;21(2):147-159. doi:10.1080/15325024.2015.1084854 27721673 PMC5051697

[39] Green BL, Saunders PA, Power E, Trauma-informed medical care: CME communication training for primary care providers. Fam Med. 2015;47(1):7-14. 25646872 PMC4316735

[40] Dorado JS, Martinez M, McArthur LE, Leibovitz T Healthy Environments And Response to Trauma in Schools (HEARTS): a whole-school, multi-level, prevention and intervention program for creating trauma-informed, safe and supportive schools. School Ment Health. 2016;8(1):163-176. doi:10.1007/s12310-016-9177-0

