# Supplementary material for: Effect of a Trauma-Awareness Course on Teachers’ Perceptions of Conflict With Preschool-Aged Children From Low-Income Urban Households: A Cluster Randomized Clinical Trial
Source: JAMA Netw Open. 2019 Apr 26;2(4):e193193. doi: 10.1001/jamanetworkopen.2019.3193 (PMC6487571; doi:10.1001/jamanetworkopen.2019.3193)
Supplement: Supplement 1. — Trial Protocol [file jamanetwopen-2-e193193-s001.pdf]

THE SCHOOL DISTRICT OF PHILADELPHIA

RESEARCH REVIEW COMMITTEE

Office of Research and Evaluation

440 North Broad Street
2nd Floor, Portal A
Philadelphia, PA 19130
Phone: 215-400-6417 Fax: 215-400-4252

Application to Conduct Research Cover Page

This is a fillable PDF.

Principal Investigator(s)/Researcher(s)

1. Last Name 1: Whitaker First Name 1: Robert Prefix 1: Dr.
2. Last Name 2: First Name 2: Prefix 2:
3. Associated Organization/Institution 1: Temple University
4. Associated Organization/Institution 2:
5. Phone Number 1: 215-707-8676 Phone Number 2: 215-707-8670
6. Email 1: rwhitaker@temple.edu Email 2:
7. Address: 3223 N. Broad Street, Suite 175
8. City: Philadelphia State: PA Zip Code: 19140

Proposed Study

9. Title of Study: A randomized controlled trial of trauma-awareness training for early childhood educators

10. Type of Study

(We only accept the following types of study proposals at this time, you may select more than one.)

- ☐ Academic Study*
- ☐ Dissertation*
- ☐ National Survey*
- ☑ Grant Mandated Evaluation
- ☑ Program Evaluation

****PLEASE NOTE**, if you selected "Academic Study," "Ph.D. Dissertation" or "National Survey," your research should relate to one of the District's Action Goals, specifically Action Goals 1, 2, or 3, outlined in Action Plan 3.0. For more information, please visit our website. If you have selected "Dissertation," please specify within your application whether it is in fulfillment of a Ph.D., Ed.D., or other degree type.*

11. If you selected **"Academic Study," "Dissertation" or "National Survey"** please list the Action Goal(s) that best describe your research.

12. If you selected **"Program Evaluation,"** please tell us the name of the program: Enhancing Trauma Awareness (ETA)

13. If you selected **"Grant Mandated Evaluation,"** please tell us the name and source of the grant: United Way of Greater Philadelphia and Southern New Jersey

Updated December 2016

14. Duration of Study **(proposals with a start date less than 6 months after submission date will incur an additional fee)**

Submission Date	08-04-17	Start Date	08-28-17	End Date	06-01-17	Final Report Submission	06-1-18

15. Identify all **District schools, divisions, and offices** involved in this research. Please include names of **contacts/collaborators,** if applicable. If you have not yet selected schools, please note key characteristics of your desired sample (e.g. "K-3rd grade students in 5 elementary schools").

The study contacts are Joy Diljohn, Executive Director of the Prekindergarten Head Start Program, and Michelle Linder-Coates, Executive Director of Bright Futures and Partnership Development.

The study will involve 128 early childhood teachers from 137 sites (centers) under the auspice of SDP (Prekindergarten Head Start [n=47], Bright Futures [n=19], and Community Partners that all operate classrooms with exclusively low-income children [n=71]).

16. Will you be requesting data from the District? ☐ Yes ☑ No

17. Do you have a standing MOU with the District? ☐ Yes ☑ No

18. If applicable, **describe the type of data you are requesting** (e.g. demographic information, test results, report card marks, school years, grades, schools, etc.). Please indicate whether you seek aggregate data or individual student records and the number of years. PLEASE NOTE, we only have student level data starting from the 2010-2011 school year.

N/A

19. In a separate document, please include a **Structured Abstract** of no more than 300 words. It will be used to communicate the essence of your research project to Administrators and others within the District who are involved in deciding whether the project aligns with District goals and needs.

Signature of Principal Investigator/Researcher 1

Signature of Principal Investigator/Researcher 2

Structured Abstract

Background. To increase school readiness, Pre-K programs for low-income children must be responsive to the role of trauma in the lives of children, families, and staff. In 2017-2018, SDP's Office of Early Childhood Education will help Pre-K teachers support children's social-emotional and behavioral health, which is essential for early learning, by offering teachers a professional development course called *Enhancing Trauma Awareness (ETA)*.

Purpose. To determine whether teachers who take ETA will have: 1) better work functioning (better quality relationships with children; greater mindfulness, empathy, emotional regulation, compassion satisfaction, and job satisfaction; and less secondary traumatic stress and burnout); 2) more trusting work relationships (with parents, supervisors, and other staff members); and 3) better health (fewer mentally and physically unhealthy days and better sleep quality/duration).

Population. Pre-K classroom teachers (n=128) working in centers under SDP auspice that serve exclusively low-income (≤300 % of poverty) children.

Intervention. A 12-week professional development course—Enhancing Trauma Awareness—will delivered by Lakeside Global Institute in 6 group sessions, with 16 teachers per group and each session lasting 2.5 hours.

Design. Consenting teachers will be randomly assigned in pairs (classroom lead and assistant) to receive the ETA course in either fall 2017 (intervention groups) or spring 2018 (wait-list control groups).

Data collection and analysis. An external evaluation team (Temple University) will administer a confidential, online survey to all 128 teachers in fall 2017 (before fall course), winter 2017 (after fall course), and spring 2018 (after spring course). Teacher-children

relationship quality will be the *a priori* primary outcome, and secondary outcomes will be assessed across the domains of work functioning, trust, and health. We will use analysis of covariance to compare means in intervention and control groups in the winter of 2017 (post-intervention) after adjusting for fall 2017 (pre-intervention) levels.

II. Proposal Sections

A. Project title: A randomized controlled trial of trauma-awareness training for early childhood educators

B. Primary investigator: Robert C. Whitaker, MD, MPH, Professor of Epidemiology and Biostatistics and Pediatrics, Temple University, 3223 North Broad Street, Suite 175, Philadelphia, PA 19140, Tel: 215-707-8676 or 215-707-8670, Email: bobwhit@temple.edu

C. Background: Exposure to adverse childhood experiences, such as abuse and neglect, is common and has life-long impacts on mental and physical health (Felitti et al., 1998; Shonkoff, Boyce, & McEwen, 2009; Shonkoff et al., 2012). These traumas affect children's ability to learn, but they also affect the functioning of teachers, many of whom can suffer from the lasting impacts of their own childhood traumas (Whitaker et al., 2014). In action Plan 3.0, the School District of Philadelphia (SDP) identified action item 7—recognize, respond to, and support students' social-emotional and behavioral health needs—which addresses 3 SDP anchor goals. Action item 7 specifies efforts to "equip staff to recognize and appropriately address students' social-emotional and behavioral needs through trauma-informed practices." The plan further acknowledges that:

> "many of our students have experienced and/or continue to experience the detrimental effects of trauma. To help mitigate the impact that these experiences have on our students' academic success, we will train our staff to understand and respond to trauma."

Present Opportunity: To address the need for trauma-informed practices in early childhood education, SDP has partnered with the United Way of Greater Philadelphia and Southern New Jersey (UWGPSNJ) and the Lakeside Global Institute (LGI) to deliver a professional development course —Enhancing Trauma Awareness (ETA)—in the 2017-2018 school year to 128 classroom teachers. These teachers work with 3- and 4-year-old children living in low-income households (≤ 300% of poverty). This includes the teachers at 137 sites (centers) under the auspice of SDP (Prekindergarten Head Start [47], Bright Futures [19], and Community Partners [71]) that all operate classrooms with exclusively low-income children. In this <u>randomized controlled trial</u>, a team at Temple University will independently evaluate the impact of ETA on the levels of work functioning, trust in work relationships, and health of Pre-K teachers. The findings will help the SDP Office of Early Childhood Education decide whether to regularly extend ETA to other teachers.

Intervention, Feasibility, and Preliminary work: ETA is delivered in 6 face-to-face group sessions (2.5 hours every other week over 12 weeks) with 16 participants per group. Classes allow participants to create a community of learning, incorporating principles of safety and nurture as the group increases trauma awareness (**Appendix B.1**). The overall goals of ETA are: 1) to increase awareness about the nature of trauma and the ways it impacts children and adults, and 2) to provide general skills for making trauma-sensitive responses to children and adults (parents and other staff) who might be trauma-impacted.

LGI has already established the fidelity and feasibility of implementing ETA. The curriculum includes an instructor's guide with a checklist for each session enumerating the topics, activities, and approaches to be used. One of the session co-instructors completes this checklist and also notes successes and challenges that occurred in the session.

Challenges are addressed in reflective supervision sessions each week with the LGI training supervisor. There are three instructor levels (apprentice, certified, mentoring) and the SDP courses will not use apprentice instructors. To reach the certified level involves serving as an apprentice instructor for 2-4 courses along with a co-instructor at the mentoring level.

We have conducted a successful pilot of ETA (**Appendix B.3**) with 16 administrative and support staff in the SDP Head Start Pre-K program, supporting the feasibility of using ETA in the proposed setting. In addition, UWGPSNJ collected preliminary evidence of the effectiveness of ETA in early childhood care and education (Jaeger & Galvano, 2013; Rind, 2014). The staff who received these courses report reacting less negatively to children's misbehavior and having more positive interactions with parents. However, there has been no random-assignment evaluation of the impact of ETA in Pre-K teachers working in classrooms with children from low-income families. Trauma awareness is especially important in these classrooms, because they serve families living in poverty, a population in which childhood trauma is more common (Corporation, 2013).

D. Research question(s): Our primary hypothesis is that ETA training will improve the quality of Pre-K teachers' relationships with the children in their classrooms (specifically, reducing levels of perceived conflict). Our secondary hypotheses are that, compared to controls, teachers who receive ETA training will also report having: 1) <u>better work functioning</u> in additional areas (greater mindfulness, empathy, emotional regulation, compassion satisfaction, and job satisfaction as well as less secondary traumatic stress and burnout); 2) <u>more trusting work relationships</u> (with parents, supervisors, and other staff members); and 3) <u>better health</u> (fewer mentally and physically unhealthy days and better quality and duration of nighttime sleep).

E. Methodology: *Experimental Design:* The ETA course will be evaluated in a randomized controlled trial to enhance causal inference about the impacts of the course on hypothesized outcomes (Shadish, Cook, & Campbell, 2002). Because SDP wishes to evaluate ETA, they have required teachers who wish to enroll in the ETA course to take the course along with their fellow classroom teacher[1] and participate in the evaluation.[2] Therefore, all who take the course must consent to be randomly assigned and complete the three evaluation surveys. We will randomly assign 64 consented teacher-pairs (128 teachers) to either the fall or spring course. The course will be taught to groups of 16 (8 teacher-pairs per group), with four groups in the fall course (32 pairs/64 teachers) and four in the spring course (32 pairs/64 teachers). To determine the impacts of the training intervention on those assigned to the fall course (intervention groups), those assigned to the spring course (control groups) will serve as concurrent controls during the fall.

Sample: The ETA course offering is intended to reach early childhood teachers serving children with the highest risk for adverse childhood experiences. Therefore, recruitment will be limited to the 137 centers under SDP auspice that serve only children in low-income households (<300% of the federal poverty level). To maximize study power, only one teacher pair per center can participate in the course. The first enrolled teacher pair in a given center will be the one randomly assigned until 64 pairs are randomly assigned.

Data Collection: Self-reported outcomes for all 128 teachers will be assessed through online surveys at 3 time points—1) fall 2017 (before the fall course), winter 2017 (after the fall course), and 3) spring 2018 (after the spring course) (**Appendix C.1**). All assessments will

[1] All early childhood classrooms have a pair of teachers, lead and assistant, and the intervention is not hypothesized to be impactful unless both teachers take it.
[2] Other course options are available that do not have these requirements.

be made through secure online surveys hosted by Qualtrics survey software. The surveys can be completed over the internet using any computer, tablet, or smartphone. There will be 4 surveys—a recruitment survey (**Appendix C.2**) and three identical evaluation surveys (**Appendix C.3**). The recruitment survey contains basic contact and demographic information obtained after online consent and will take less than 5 minutes to complete. The 3 evaluation surveys will each take 30 minutes to complete. They assess constructs in three major domains—work functioning, work relationships, and health (see table on page 1 of **Appendix C.3** for information on validated items and scales). [3] Attendance data at each session will be collected by the LGI and provided to the Temple research team.

F. Logic model and evaluation plan:

Logic Model for Trauma Awareness Course for SDP Early Childhood Teachers

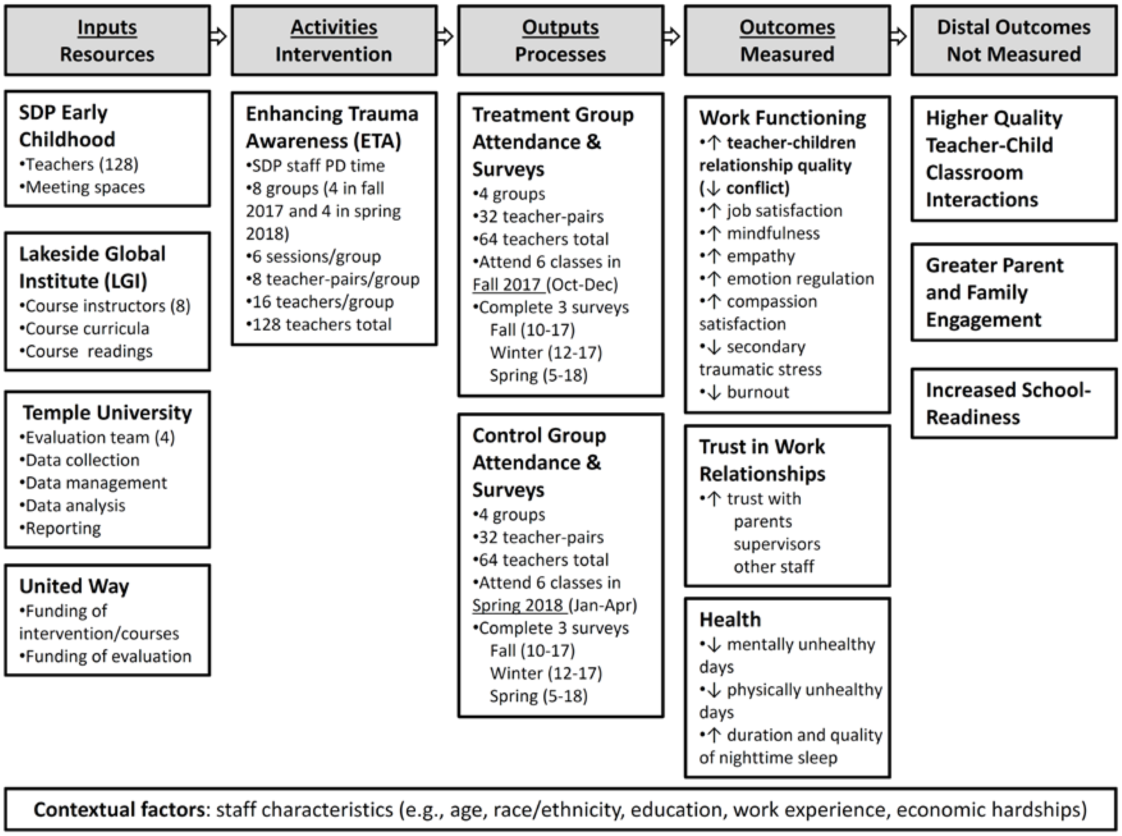

---

[3] The third and final evaluation survey differs slightly in that it also asks participants about their own adverse childhood experiences. These data are important data for describing the study participants and determining the external validity or generalizability of the evaluation findings.

Data Collected and Analyses: This random-assignment evaluation of ETA involves assessing impacts in 3 outcome domains— work functioning, trust in work relationships, and health—by comparing intervention and control group means of measures in each domain (**Logic Model and Appendix C.3, page 1**). We will first confirm that the distributions of the outcome variables conform to the assumptions of our analytic models. To evaluate the effect of our random assignment, we will compare the sociodemographic characteristics of the intervention and control groups using t-tests and chi-square tests. We will also report means and standard deviations from assessments in all three surveys. We will use an ordinary least squares analysis of covariance (ANCOVA) to evaluate the intervention effects by comparing follow-up (winter 2017) means in the intervention and control groups in an intention-to treat analysis. The ANCOVA model for each outcome will include weighted contributions of the teacher's pre-intervention score (fall 2017) and intervention/control status, weighted contributions of the classroom's average pre-intervention score, and teacher- and classroom-specific error terms. We add the classroom average pre-intervention score to improve precision in the intervention effect (Bloom, Richburg-Hayes, & Black, 2007). The significance of the intervention will be determined by a statistically significant (alpha =.05) coefficient for the intervention/control status indicator.

Study Power: Using Optimal Design software (Spybrook et al., 2011), we conducted a power analysis for the multi-level treatment estimate assuming that variation in pre-intervention (fall 2017) scores accounts for 50% of the variation in post-intervention (winter 2017 scores. Given the number of planned participants (n=128 teachers or 64 pairs), clustering at the classroom level (2 teachers per classroom), an alpha of .05, and a power of 80%, the

minimal detectable effect (MDE) size will be 0.50. If there are only 116 teachers at follow-up, the MDE would rise to 0.53.

G. Benefit of study to participants and to District: There are no direct benefits to the study participants. However, both the participants and SDP may benefit from knowing that the information they provide will support the SDP Office of Early Childhood Education in: 1) addressing action item 7 (and related anchor goals) in the SDP Action Plan 3.0, and 2) accumulating evidence about whether the ETA course is achieving its desired impacts and might benefit other SDP early childhood teachers. Participants' will be compensated up to $90 for this time spent outside of work hours completing the 3 evaluation surveys—a $30 electronic Amazon gift card will be sent by email after each completed online survey.

H. Burden on study participants and on District: Respondent risks are minimal. There are some survey questions that some respondents might find stressful and there is a potential risk of loss of confidentiality if electronic data are not securely held. Safeguards against these risks are discussed below. SDP and participant burdens are also minimal. The ETA course sessions will occur on Friday afternoons during regular work hours when staff are already being compensated. On Friday afternoons, most Pre-K teachers (Pre-K Head Start and Bright Futures) have designated class preparation time. Substitute teachers will be provided for teachers from community partner sites who take the ETA course. The time burden for the evaluation will be limited to 90 minutes (three online surveys of 30 minutes each). No data are being requested from SDP.

I. Compliance with federal regulations: To recruit teachers, the SDP early childhood executive directors (Diljohn and Miller-Coates, **see Appendix F**) will use email to disseminate information about the ETA course offering to center directors and teachers, all

of whom have email accounts to receive work-related announcements. This email will include information describing the evaluation study (**Appendix B.4**). A link will be provided in the email to allow teachers potentially interested in the ETA course to provide secure online consent for participation in the course and its evaluation (**Appendix A**). Those who consent to participate will also complete a brief online recruitment survey (**Appendix C.2**) that provides basic socio-demographic data along with necessary contact information for completing the 3 evaluations surveys (**Appendix C.3**).

There will be no student contact (FERPA and PPRA not applicable) and no collection of protected health information (HIPAA not applicable). The survey contains some sensitive questions about stressful experiences, such as childhood adversities and current stressors at work. Participants will be free to skip any questions they do not wish to answer. The ETA courses will be led by highly-trained instructors from LGI who have experience supporting course participants as they may become aware of the role of traumatic stress in their own lives, both past and present. The support provided by the instructors, as well as by other group members, is a central feature of the courses. As needed, all of the instructors will also be able to connect participants to additional mental health resources in the community.

Appendix B.2 provides details on the protocol, approved by Temple IRB, to protect privacy and confidentiality. In brief, all personally identifiable information will be kept in a linkage data file separate from the de-identified analytic data file, and these two files will be linked only by an anonymous study ID. We will retain the de-identified analytic data file indefinitely to assure the integrity of the data analysis as part of the peer review process. The electronic linkage data file will be kept for a period of 60 months after the end of the

data collection and then be destroyed. To protect against the possibility of deductive disclosure, data will be reported in aggregate. Access to both the analytic and linkage data files will be restricted to Temple University research staff, and both files will be maintained at all times on a secure server.

J. Plan for dissemination of findings: We will summarize the findings in the form of a report suitable for submission to a peer-reviewed scientific journal (e.g., *Early Childhood Research Quarterly*, *Early Education and Development*, *Child Development Perspectives*, *Journal of Traumatic Stress*). We will share our results with SDP staff during an oral presentation, and we will also present the results in an oral or poster presentation at a national conference, such as at the Society for Research in Child Development Biennial Meeting, the Society for Research on Educational Effectiveness Annual Meeting, or the National Research Conference on Early Childhood. All partners in this project—SDP, UWGPSNJ, LGI, and Temple University (**Logic Model**) — will continue dialogue during and after the research process about how the information obtained in the evaluation can help inform ongoing efforts by SDP to develop trauma-responsive early childhood education.

K. Reference List

Bloom, H. S., Richburg-Hayes, L., & Black, A. R. (2007). Using covariates to improve precision for studies that randomize schools to evaluate educational interventions. Educational Evaluation and Policy Analysis, 29(1), 30-59. doi:doi:10.3102/0162373707299550

Felitti, V. J., Anda, R. F., Nordenberg, D., Williamson, D. F., Spitz, A. M., Edwards, V., . . . Marks, J. S. (1998). Relationship of childhood abuse and household dysfunction to many of the leading causes of death in adults: The Adverse Childhood Experiences (ACE) Study. *American Journal of Preventive Medicine, 14*(4), 245-258.

Jaeger, E., & Galvano, L. (2013). Opening minds, opening hearts: Final report of the evaluation of the Institute for Family Professionals trauma courses offered through Saint Joseph's University. Philadelphia, PA: United Way of Greater Philadelphia and Southern New Jersey.

McCartney, K. (2000). Effect size, practical importance, and social policy for children. *Child Development, 71*(1), 173-180. doi: 10.1111/1467-8624.00131

Public Health Managment Corporation. (2013). *Findings from the Philadelphia Urban ACE Survey*. Retrieved from http://www.instituteforsafefamilies.org/sites/default/files/isfFiles/Philadelphia%20Urban%20ACE%20Report%202013.pdf.

Rind, B. (2014). An empirical examination of the impact of Institute for Family Professionals courses: Full report. Philadelphia, PA: United Way of Greater Philadelphia and Southern New Jersey.

Shadish, W. R., Cook, T. D., & Campbell, D. T. (2002). *Experimental and quasi-experimental designs for generalized causal inference*. Boston, MA: Houghton Mifflin Company.

Shonkoff, J. P., Boyce, W. T., & McEwen, B. S. (2009). Neuroscience, molecular biology, and the childhood roots of health disparities: Building a new framework for health promotion and disease prevention. *Journal of the American Medical Association, 301*(21), 2252-2259. doi:10.1001/jama.2009.754

Shonkoff, J. P., Garner, A. S., Siegel, B. S., Dobbins, M. I., Earls, M. F., McGuinn, L., . . . Wood, D. L. (2012). The lifelong effects of early childhood adversity and toxic stress. *Pediatrics, 129*(1), e232-e246. doi: 10.1542/peds.2011-2663

Spybrook, J., Bloom, H., Congdon, R., Hill, C., Martinez, A., & Raudenbush, S. W. (2011). Optimal Design Plus Empirical Evidence: Documentation for the "Optimal Design" Software Version 3.0. Retrieved from http://wtgrantfoundation.org/resource/optimal-design-with-empirical-information-od

Whitaker, R. C., Dearth-Wesley, T., Gooze, R. A., Becker, B. D., Gallagher, K. C., & McEwen, B. S. (2014). Adverse childhood experiences, dispositional mindfulness, and adult health. *Preventive Medicine, 67*, 147-153. doi: 10.1016/j.ypmed.2014.07.029

School District of Philadelphia
Office of Research and Evaluation

Amendment Form for Approved Research

1. Name: Robert C. Whitaker

2. Date of Amendment Request Submission: August 21, 2017

3. Organization: Temple University

4. Name of Primary Researcher on Approved Study: Robert C. Whitaker

5. Phone: 215-707-8676

6. Email: rwhitaker@temple.edu

7. Study Title: A randomized controlled trial of trauma-awareness training for early childhood educators

8. Study #: Still under review

9. Describe in detail the proposed amendments you would like to make to your study and the date they would go into effect:

We are adding the opportunity for additional compensation. In addition to participants receiving a $30 Amazon gift card for each completed survey (outlined in the original protocol), there will be three lotteries, with each lottery offering two $100 gift cards. Participants completing evaluation survey 1 will be entered into lottery 1 in September 2017. Participants completing evaluation surveys 1 and 2 will be entered into lottery 2 in December 2017. Participants completing evaluation surveys 1, 2, and 3 will be entered into lottery 3 in May 2018.

10. Explain in detail why these changes are necessary:

We decided to add the lottery incentive in order to boost recruitment of participants, as well as increase the likelihood that participants will complete all three evaluation surveys, given the duration of the study.

11. Are you submitting additional documents with you amendment?

Yes ☑ No ☐

And they include:

We are attaching clean versions of a revised research proposal, consent (Appendix A), a
