# Supplementary material for: Effect of a Trauma-Awareness Course on Teachers’ Perceptions of Conflict With Preschool-Aged Children From Low-Income Urban Households: A Cluster Randomized Clinical Trial
Source: JAMA Netw Open. 2019 Apr 26;2(4):e193193. doi: 10.1001/jamanetworkopen.2019.3193 (PMC6487571; doi:10.1001/jamanetworkopen.2019.3193)
Supplement: Supplement 2. — eFigure. Conceptual Framework Guiding the Evaluation of Enhancing Trauma Awareness eAppendix 1. Description of the Enhancing Trauma Awareness Course eAppendix 2. Focus Group Methods for Evaluating the Enhancing Trauma Awareness Course eAppendix 3. Qualitative Themes Supporting Fidelity to the Enhancing Trauma Awareness Course Content and Relational Process eTable 1. Detailed Instrument Descriptions for the Trial Outcome Measures eTable 2. Neighborhood Characteristics of Eligible Early Childhood Education Sites, by Participation in the Trial eTable 3. Subgroup Comparisons of the Effect of Enhancing Trauma Awareness on Teacher-Children Conflict Scores eTable 4. Effect of Enhancing Trauma Awareness at Delayed Follow-up on Measures of Relationship Quality, Relational Capacities, and Health and Well-being eTable 5. Changes in Outcome Measures Between Baseline and Delayed Follow-up for Those in the Intervention Group Who Did and Did Not Participate in Focus Groups eTable 6. Additional Teacher Focus Group Quotes Supporting Outcomes in the Conceptual Framework eReferences [file jamanetwopen-2-e193193-s002.pdf]

Supplementary Online Content

Whitaker RC, Herman AN, Dearth-Wesley T, et al. Effect of a trauma-awareness course on teachers' perceptions of conflict with preschool-aged children from low-income urban households: a cluster randomized clinical trial. *JAMA Netw Open.* 2019;2(4):e193193. doi:10.1001/jamanetworkopen.2019.3193

eFigure. Conceptual Framework Guiding the Evaluation of Enhancing Trauma Awareness

eAppendix 1. Description of the Enhancing Trauma Awareness Course

eAppendix 2. Focus Group Methods for Evaluating the Enhancing Trauma Awareness Course

eAppendix 3. Qualitative Themes Supporting Fidelity to the Enhancing Trauma Awareness Course Content and Relational Process

eTable 1. Detailed Instrument Descriptions for the Trial Outcome Measures

eTable 2. Neighborhood Characteristics of Eligible Early Childhood Education Sites, by Participation in the Trial

eTable 3. Subgroup Comparisons of the Effect of Enhancing Trauma Awareness on Teacher-Children Conflict Scores

eTable 4. Effect of Enhancing Trauma Awareness at Delayed Follow-up on Measures of Relationship Quality, Relational Capacities, and Health and Well-being

eTable 5. Changes in Outcome Measures Between Baseline and Delayed Follow-up for Those in the Intervention Group Who Did and Did Not Participate in Focus Groups

eTable 6. Additional Teacher Focus Group Quotes Supporting Outcomes in the Conceptual Framework

eReferences

This supplementary material has been provided by the authors to give readers additional information about their work.

© 2019 Whitaker RC et al. *JAMA Network Open.*

eFigure. Conceptual Framework Guiding the Evaluation of Enhancing Trauma Awareness

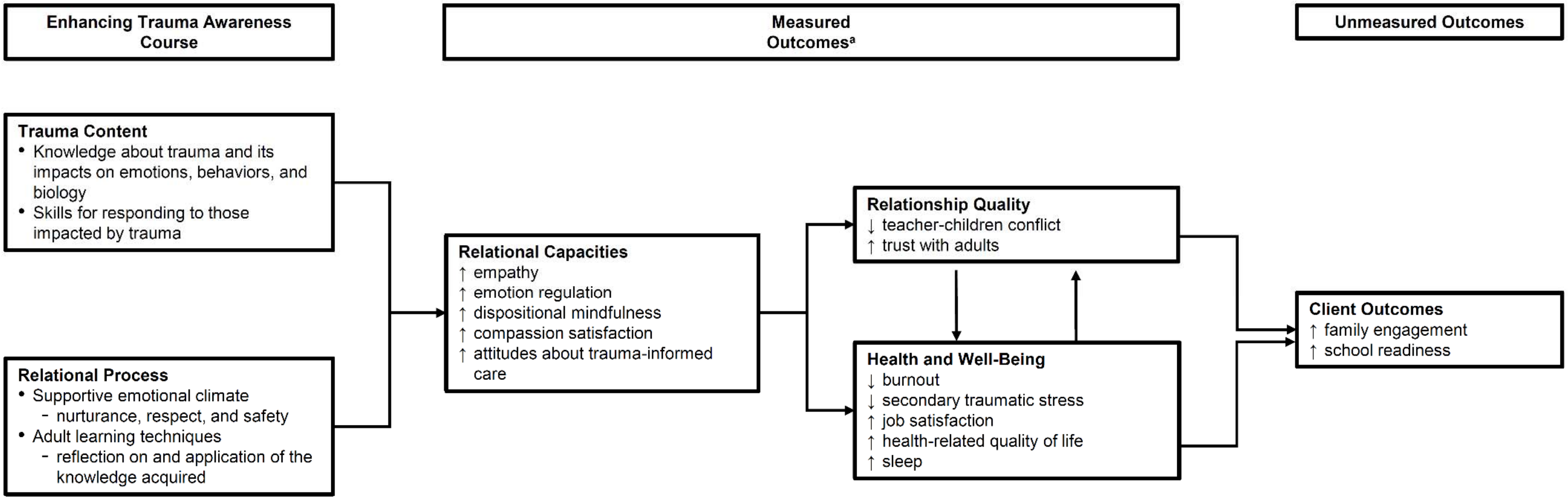

[a] ↑↓ arrows signify the hypothesized direction of the impact of the course on the outcome measure

© 2019 Whitaker RC et al. *JAMA Network Open.*

eAppendix 1. Description of the Enhancing Trauma Awareness Course

A. Overview

Enhancing Trauma Awareness was designed by Lakeside Global Institute (North Wales, PA) for professionals, such as those in healthcare, human services, and education, who work with people who may have experienced trauma. The study authors were independent evaluators who were not involved in the development or delivery of ETA.

The course is intended to help the course participants to:

- increase their awareness and understanding of the impacts of trauma in the lives of those they serve, as well as in their own lives;
- shift their attitudes and perceptions about trauma and its consequences toward being more trauma-responsive;
- better manage trauma-related emotions and behaviors in themselves and others; and
- build trusting relationships with colleagues and those they serve.

B. Trauma content (summarized from the following reference)[1] (see references for course reading materials)[2, 3]

Session 1

- Defining trauma, post-traumatic stress disorder (PTSD), and trauma-triggers
- Similarities and differences among the following terms: trauma-aware, trauma-sensitive, trauma-competent, and trauma-informed services
- Use of the term trauma-impacted person vs. trauma victim or trauma survivor
- Potential for the study of trauma to be traumatic and the need for personal strategies (safety plans) to remain emotionally safe while studying trauma

Session 2

- Key parts of the brain involved in trauma and how the parts work together
- Possible actions of trauma-aware and -informed professionals: prevent trauma, avoid triggering those with unresolved trauma, respond sensitively to trauma-impacted people, use appropriate trauma therapies to promote healing

Session 3

- Adverse childhood experiences and their consequences, including the trans-generational transmission of trauma
- Trauma as a sensory experience that can remain dormant until triggered
- Traumatic reenactment or repetition
- Brain states in response to trauma: the continuum from being calm to being alert/aroused, alarmed, fearful, and terrorized
- Brain-based responses to trauma: dissociation and hyper-arousal
- Potential for positive impacts of trauma

Session 4

- Dissociation, somatization, and affect dysregulation as adaptations to trauma
- Differences between grief and trauma
- The role of memory in trauma and trauma-triggers
- PTSD and complex trauma in early childhood
- Initial responses to trauma: establishing physical and emotional safety before de-briefing or disclosure
- Somatic therapies to address trauma
- Capacity to recover from trauma without psycho-therapeutic interventions

© 2019 Whitaker RC et al. *JAMA Network Open.*

Session 5

- Differences among the following terms: recovery, resolution, restoration, reconcile, repair, and healing
- Stage-based approach to trauma with safety as the foundation for healing
- Risk and protective factors that modify the impact of potentially traumatic experiences
- Two major categories of trauma (circumstantial and relational) and definitions of types of trauma (e.g., vicarious, developmental, complex)

Session 6

- Needs of trauma-impacted people and ways to address needs
- Needs of those who interact with trauma-impacted people and ways to address needs
- Ways to promote physical and emotional safety for trauma-impacted people

C. Relational processes

Techniques of adult learning

- Provide opportunities to apply knowledge to address current challenges at work and outside of work
- Provide opportunities to reflect on the knowledge acquired and its impacts (e.g., reflective sharing in class, discussion of course readings, and reflective writing on course readings)
- Use participants' professional and other life experiences as a resource for learning
- Use stories, analogies, images, and demonstrations
- Have instructors act as facilitators of learning

Techniques for creating a supportive emotional climate that helps course participants feel safe, respected, and nurtured

- Use two (co-) instructors in each session
- Review communication "ground rules" before each session (e.g., no side conversations, cell phones, or monopolizing time; maintain confidentiality)
- Develop and support the use of personal "safety plans" to prevent the study of trauma from being traumatizing
- Allow participants to excuse themselves from an activity or discussion if they feel unsafe or triggered
- Provide predictions about what may happen to participants' understanding and emotions as they move through the course
- Provide disclaimers about what instructors cannot present or accomplish
- Provide a message of hope about recovery and healing after trauma
- Use and model (with co-instructor and participants) active listening techniques, non-judgmental attitude and communication style, and trauma-responsive behaviors
- Show appreciation for participants' professional and other life experiences
- Avoid blaming or shaming
- Accept and validate a range of emotions and experiences
- Respond to participants' suggestions and needs
- Do not grade or evaluate
- Provide food
- Start and end on time

© 2019 Whitaker RC et al. *JAMA Network Open.*

D. Trainer preparation

The ETA curriculum was developed by L. Diane Wagenhals, M.Ed., who also developed the process at Lakeside Global Institute (LGI) (North Wales, PA) for certifying trainers who teach the ETA course. There are three trainer levels—apprentice, certified, and mentoring. All nine trainers in the randomized trial of ETA were at the certified or mentoring level.

LGI offers three sequential trauma-focused courses, resulting in a certification from LGI that the participant is Trauma Competent. ETA is the first of the three courses and is followed by Deepening Trauma Awareness (DTA) and Applying Trauma Principles (ATP). Both DTA and ATP consist of 12, 2.5 hour sessions offered over 24 weeks.

Potential trainers are identified by certified or mentoring trainers based on their demonstration of leadership skills as they move through the sequence of courses. In addition to completing the three trauma courses, potential trainers must also complete the Group Facilitation course and Essential Communications course. Trainers for these two courses also submit recommendations for potential trainers. Those who decide to become apprentice trainers must then complete a three-session Leadership Training Course, pass a written exam, and receive the recommendation of the two course leaders.

To become certified, the apprentice trainer must then lead two to four ETA courses with a co-trainer who is at the mentoring level, a level designated for the most experienced certified trainers. Certification is granted after the apprentice trainer has demonstrated adequate competency while leading these ETA courses with the mentoring trainer. Of the 9 trainers in the trial, 6 were older than 50 years of age, 7 were female, 6 were non-Hispanic white, and 6 had master's degrees. Five of the trainers had taught ETA 25 or more times (range 4-30).

E. ETA quality control

Fidelity of course implementation is supported by the use of a detailed trainer's guide, and after each session, the co-trainers complete a checklist enumerating the topics, activities, and approaches used. During any ETA course, the co-trainers discuss any challenges occurring while leading the course. In addition, each trainer has monthly one-on-one reflective supervision sessions with their training supervisor in which challenges, questions, and successes are processed and feedback is given. All trainers also complete a Trainer Evaluation Form for each session in which they share their successes and challenges for that session. Each of these forms is submitted and reviewed by the LGI director of administration and the program director. At a monthly meeting of all course trainers at LGI, the trainers describe and process their previous month's experiences in the courses they are teaching and receive feedback from their colleagues, supervisors, and the program director.

© 2019 Whitaker RC et al. *JAMA Network Open.*

eAppendix 2. Focus Group Methods for Evaluating the Enhancing Trauma Awareness Course

A. Participants

Of the 48 teachers allocated to the intervention group in the randomized trial, the 38 who attended at least four of the six ETA course sessions (fall 2017) were eligible to participate in one of two focus groups held in May 2018, approximately five months after the end of the course. We contacted the 38 eligible teachers by email, telephone, and text messages with the aim of conducting two focus groups with up to 12 participants in each group. We were able to recruit a total of 15 teachers, with 8 participating in one focus group and 7 in another focus group.

In June 2018, we conducted a focus group with the trainers who taught the ETA course during the intervention period (fall 2017). The trainers were contacted by email about their interest in participating in a focus group, and all 9 trainers agreed to participate. This included the two trainers assigned to each of the four course locations and the one trainer who served as the available substitute.

The protocols for the focus group data collection and analysis were approved by the Institutional Review Board of the Mary Imogene Bassett Hospital (Cooperstown, NY). All focus group participants provided written informed consent. At the conclusion of each focus group, participants received a $100 Amazon gift card to compensate them for their time and any transportation or family care costs.

B. Procedures

The two teacher focus groups were held in a conference room at the School District of Philadelphia's administrative office. The trainer focus group was held in a conference room at Lakeside Global Institute (North Wales, PA) where the trainers were employed. Two of us (ANH and RCW), who are experienced facilitators, led the groups. Each focus group lasted approximately 90 minutes and was digitally audio and video recorded. Before the trainer focus group was started, each of the nine trainers completed a brief demographic survey. The overarching goal of the focus groups was to understand how the early childhood education teachers (ETA course participants) and trainers experienced the ETA course, especially the intrapersonal and interpersonal processes and outcomes that might not be captured by the close-ended questionnaires completed by the trial participants. Specifically, we were interested in whether and how, as a result of taking the course, the teachers experienced any changes in the three major outcome domains of the trial (eFigure)—relational capacities, health and well-being, and the quality of their relationships. For the trainers, we were interested in how they experienced teaching the course to this specific group (early childhood teachers working with children in low-income households) in comparison to other groups they had taught. To address these areas, separate focus group guides, with open-ended questions, were developed for the teachers and the trainers (sections D and E below). The questions avoided suggesting hypothesized outcomes or particulars of the course content or process.

C. Analysis

Three team members (SBB, ELM, and AMS) transcribed the focus group recordings verbatim and checked the accuracy of each other's transcriptions. Each of these three team members then independently generated a list of codes (i.e., recurring concepts or ideas in the transcripts) for the two teacher focus groups and a separate code list for the trainer focus group (6 total code lists). This was done as an inductive process, without using any predetermined set of constructs, such as those in the conceptual framework that guided selection of the questionnaires used in the quantitative part of the impact evaluation (eFigure). Using these six independently generated code lists, the three coders, together with another of us (ANH), identified similar codes across the lists and compiled a single consensus code list for the two teacher focus groups and a single consensus code list for the trainer focus group, achieving consensus about the name of each code and a brief description of its scope. Using these consensus codes lists (20 codes in the teacher list and 15 in the trainer list), two team members (SBB and AMS) independently coded each focus group, applying up to four of the consensus codes per utterance, as appropriate, and leaving utterances without a code if none of the codes applied. Three team members (ANH, SBB, and AMS) then reviewed each coded utterance to achieve consensus about which code(s) should apply to each utterance in those instances where there was not agreement.

© 2019 Whitaker RC et al. *JAMA Network Open.*

For each code, we tabulated the number of focus group participants who made at least one utterance to which that code was applied. For the teacher focus groups, we considered any code to be a theme if 8 or more of the 15 participating teachers had an utterance to which that code was applied. Similarly, for the trainer focus group, we considered any code to be a theme if 5 or more of the 9 participating trainers had an utterance to which that code was applied. Applying these criteria resulted in 11 teacher themes and 10 trainer themes. For the purposes of this report, we (RCW and ANH) then identified the teacher themes that supported one or more outcome constructs in our conceptual framework (eFigure). We paid particular attention to spontaneous reports by the 15 teachers in the focus groups of changes in their behaviors with children that resulted from taking the ETA course. Because teachers were not masked to the intervention, we attempted to avoid reporting bias in the trial by not including self-reported behaviors as part of the questionnaire-based assessments. We (RCW and ANH) also identified themes from both the teacher and trainer focus groups that supported fidelity of the implementation of the ETA curriculum with regard to trauma content or relational process.

D. Focus group guide for course participants (early childhood teachers)

Impacts of the course on perceptions and attitudes

- What do you remember most about the course? Can you think of something about the course that most captured your interest or attention? Why?
- In what ways was this course different than what you expected? Did anything surprise you? Was anything more difficult than you expected?
- What did you learn in ETA? By "learn," we mean ways of thinking about things that were new to you.
- Was this the sort of class in which you ended up saying more than you expected? Less than you expected? Why?
- Talk to us a bit about your course instructors.
- What did you learn about your classmates? About yourself?
- Because the topic of trauma can be emotional, we imagine that being in this course could bring up a lot of feelings, maybe some good or maybe some bad. How did you feel during the course?

Impacts of the course on behaviors

- Has anything in the course changed your day-to-day life at work? If yes, why? If not, why not?
    - (If there is a more emotion-based response) Can you provide some concrete examples of what that looks like at work?
    - (If there is a more behaviorally-based response) What motivated you to do that?
- Has anything in the course changed your day-to-day life outside of work? If yes, why? If not, why not?
    - (If there is a more emotion-based response) Can you provide some concrete examples of what that looks like in your life?
    - (If there is a more behaviorally-based response) What motivated you to do that?
- Have you shared anything about the course with friends, family members, colleagues, or others in your life? If you did, why did you share it?
- Is there anything else you wished to share about your experience taking the course that you did not get a chance to say?

E. Focus group guide for trainers

Experiences teaching the course to this population

You have taught many ETA courses. We are curious about your experiences with this group of early childhood educators and how this might compare to participants in past ETA courses you have taught.

- Tell us about your experience teaching the course with these early childhood educators.
- While teaching the course, what did you come to understand about the trauma histories of these course participants? How, if at all, did their experiences affect the way you taught the course?

Group engagement

ETA is designed as a group process that relies on engagement of the course participants.
(Of note, for the first three bulleted questions below, consider probes about whether the success of the group, relationships to other participants, and relationships between trainer and participant felt similar or different to other ETA groups.)

- What does a “successful” ETA group feel like to you? Did your group with the early childhood teachers feel “successful”? If so, why? If not, why not?
- How would you describe the relationships that were formed in the group?
- How would you describe your relationships with these participants?
- What, if anything, do you think children might notice had changed because their teachers were in your course?

Placing the group in context

Groups of ETA course participants vary in their prior knowledge and understanding of trauma and how it impacts people’s lives. We want to understand your impressions of this group compared to the “average” group taking the ETA course.

- Tell me about this group’s prior knowledge of trauma and how trauma impacts people’s lives. How does this group compare to the “average” group taking the ETA course?
- As a result of the course, where do you think these teachers ended up in terms of their trauma awareness? How does this compare to the “average” group taking the ETA course?
- Did this group share more or less than the “average” group taking the ETA course? How about personal trauma, did they share more or less? How about their clients’ trauma, did they share more or less?

© 2019 Whitaker RC et al. *JAMA Network Open.*

eAppendix 3. Qualitative Themes Supporting Fidelity to the Enhancing Trauma Awareness Course Content and Relational Process

Model Construct:
Trauma content: Knowledge about trauma

Qualitative Themes:
Participant Theme 4: Greater knowledge of different types of trauma (9 speakers)[1]
Participant Theme 6: Greater understanding of personal experiences as traumatic (10 speakers)
Trainer Theme 5: Participants had significant past trauma (8 speakers)[2]

Example Quotes:
"I think one of the wonderful things is when they [course participants] do reflections because oftentimes they may not say all that they want to say, in the space that's there, and in one of the classes there was just a lot of reflection, about this person's mother [who] had passed a long time ago, but because they were together, like the teacher and the assistant teacher, I was able to make reflections on that. Then it was close to the end where the assistant teacher said, 'Oh my gosh. Thank you so much for doing that for her. She was able to talk to me for the first time, we've worked together for twelve years, this is something that has been so incredibly hard, and she didn't understand that this was trauma and that the way that it happened and all of that, and it makes sense to her now.' And then she also started talking about the fact that she kept it all in, and what it was even doing to her and to her classroom and everything else, so for us to open that up to talk about it allowed her [to be] forever changed. I just think that not having that opportunity to be able to be there, to even slowly do that through the writings, and then have someone else that was able to talk to you, I just thought was so incredible, and to understand that it makes sense to feel the way that you do and that it impacted everything, including the work that she did for these young children." (Trainer 6, FG3,[3] Trainer Themes 5 and 9)

"It [the ETA course] was so eye opening…I had no idea when I signed up for the course, like how deep it was gonna get. I remember after the first survey, and then going to the class, the first time reading some of the materials, it just took me to a lot of places personally in my life, in my past. I have two daughters, like with my children, and even my mother, like just thinking about the intergenerational piece that is talked about. And I'm having a different understanding of that and I was really grateful to be part of our group that was able to share and connect that way." (Participant 4, FG2, Participant Themes 2 and 6)

Participant 6, FG1: "You think this is trauma [shaking hands in the air]."
Participant 8, FG1: "We thought trauma had to be something so devastating, and it doesn't have to be."
Participant 6, FG1: "And it doesn't have to be."
Participant 7, FG1: "Right, it doesn't have to be. Because you have like, the alcoholism, the drug addiction now, all that is traumatizing to kids, you know, the kids are bringing drugs to school, kids are [saying] my mom sleeps all the time when I go home." (Participant Theme 4)

"Initially, the idea of getting out from work a little bit [while taking the ETA course], getting away from the kids, it was just exciting. But when I actually got to the course, I was like, hold up, I'm gonna have to really put some work into it, it felt like a college course at first…And I remember having all these thoughts in my head, like that this book said that, this book said this, and things that really stood out to me. Now, I have conversations with people about things that people are doing, that are perceived as net negative. I'm always telling people, you just really don't know what's happening, like everybody's a victim, like there's always two victims, it's never just one victim. The person that's being victimized, and the person doing the ill act, you have to look at that person, like what made that person do that. You can't just assume that everybody has ill intentions all the time or they want to be that way. Because trauma is real, and I feel like if people, especially looking at society, [if] they could see what's happening in the

---

1. Indicates the number of focus group participants (out of 15 teachers) who had at least one comment during the focus group that supported the theme.
2. Indicates the number of focus group participants (out of 9 trainers) who had at least one comment during the focus group that supported the theme.
3. FG = focus group

© 2019 Whitaker RC et al. *JAMA Network Open.*

media and such, if you really understood trauma and understand why people are doing these things, then the world would be such a better place. A lot of people don't see trauma as a real thing though, and because of that there are people that are being discarded, not considered, not being taken care of because they have a trauma that's leading to risky behaviors and mental illness and such. So yeah it was real powerful, and I didn't expect that it was going to get that deep." (Participant 6, FG2, Participant Theme 4)

Model Construct:
Trauma content: Skills for responding to trauma

Qualitative Themes:
Participant Theme 9: More mindful of trauma in managing the classroom (14 speakers)
Trainer Theme 9: Changed participants' behaviors (8 speakers)

Example Quotes:
"Because it [referencing an earlier quote regarding how the ETA course helped her to be more sensitive and calm] made me more aware of what I was doing. Should you do that with that child? Should you say that to that child? Should you act that way with that child, you know? That's a child, that's not an adult, that's not a teenager…so it taught me how to come down, come out of myself. And bring my emotions down, to deal with the smaller ones…" (Participant 6, FG1, Participant Theme 9)

"I think it's also important to point out the integration of the information, because of the way our process is set up, the frequency of the classes being two weeks apart, and then they go take the information, and they try it in real life situations, and they come back and they process through that. There seems to be a deeper level of integration. The feedback that we get is that a lot of the other trauma information they get doesn't stick with them as well as it does from our program because of the opportunities to get the information integrated, try it out, process it, come back, reflect on it, come back. And that whole process is so rich for people who are trying to change the way they do things, and so I feel so honored to be with groups, knowing that after six sessions that they have changed…they're pretty confident that they're starting to respond differently and that this is going to stick with them. It's not going to be something where, 'while I was here, it sounded good, but I'm not able to really figure out how to use it,' because they've been doing it for that long period of time." (Trainer 3, FG3, Trainer Theme 9)

"When they shift from saying to themselves, what's your problem, to what happened to you, and when they say that, and then they respond differently, and when you hear them say there was one time when a child did this, I looked at it differently, I considered the iceberg [an analogy presented in the ETA course, to suggest that emotions arising from past experiences may be occurring below the surface of an individual's outward behavior]. When the parents came to the class and were saying so-and-so, I thought to myself, what happened, so when there is a shift, and when they verbalize that, I used to do this, but now I wonder what happened, that is the success. It's a change from the inside going to the outside, changing your self-talk, which then changes your behavior and makes such a big difference in one's response, building that relationship bridge, which is what this is all about." (Trainer 7, FG3, Trainer Theme 9)

"And I think one of the unique things about this community is many educators, but especially with younger kids, is a lot of the challenging behaviors can be triggering, from the moment they get involved…literally from the moment they walk into work to the moment they leave, and I think many of these teachers felt that type of thing. I think the whole brain mirror neuron piece of, I need to be calm myself and I need to take care of myself, have strategies for myself in the moment and also self-care strategies, so that I'm in a different place when I respond and try to meet the needs of the kids, and I think the kids were really picking up on that, from the stories that they were bringing back and a lot of the reflective processing, was that my teacher was a lot calmer in terms of responding to my needs." (Trainer 3, FG3, Trainer Theme 9)

Model Construct:
Relational process: supportive emotional climate

Qualitative Theme(s):
Participant Theme 1: Experienced safety during the course (11 speakers)
Trainer Theme 1: Trainers provided safety (8 speakers)

© 2019 Whitaker RC et al. *JAMA Network Open.*

Trainer Theme 2: Trainers provided nurturance (9 speakers)
Trainer Theme 3: Trainers validated participants' experiences (8 speakers)

Example Quotes:
"Well I think there's a lot of levels to it. One of the first ones that's set up is that there's food available. They're coming from [their] classrooms [with children], they travelled far to get there, and so they get there and to be able to eat and feel nourished in that sense, so that's the one piece that I think is very tangible. But what we actually do in class and how we are in class is that we listen, it's completely non-judgmental, we are open to all opinions and perspectives, and we set really clear ground rules for safety, so people can ease into that, and that safety translates to nurturing." (Trainer 5, FG3, Trainer Themes 1 and 2)

"…oftentimes I feel that the teachers [participating] in our [ETA] class felt like they were almost an island unto themselves. The people above them didn't really understand them or appreciate them. I think one of the big things we do is that we all believe just how important early childhood education is, and so we convey that message to them, that they're building the brains, really of the future, and it really has an incredible impact on them that they feel. And just the subject of trauma, that it's never recognized that the children they're teaching are trauma-impacted, and that it's never recognized that they may be suffering from vicarious trauma because they work in these communities, and oftentimes the teachers come from these communities. So, a lot of it is stuff that they already know, but when we lift them up, and we validate that, they're just like, 'oh, now science and these people are coming, and they believe in me, and they're validating the things that I thought I knew.' A light goes on for them, and so they begin to create more validating environments for the children they work with, which is a powerful, kinda trickle-down." (Trainer 9, FG3, Trainer Themes 3 and 9)

"…we [teachers taking the ETA course] knew each other and didn't know each other, and the first thing they [ETA trainers] did was make it a safe place…and you began to feel that you could express things. Even with your family you know how certain members of your family are, you don't wanna tell them certain things in certain ways but when you have, I remember now it's coming back to me, that we're not gonna judge anybody, you got a chance to tell your story. And it wasn't their job to solve the problem or comment on it. You could safely tell your story. And to be able to be open…so just being able to be in the group to share this and to share these different things, with other people is, like you said to be able to talk and let people know how you really feel about things. And you're not being judged on it, you're just saying what you feel, was very cathartic. You know what I'm saying? It just made you understand, and then somebody else said something that they're feeling. It just made everybody get together and, like you said, talk. They're human. We all have these experiences and things we don't have control over and that's what was really good about it to know, I'm going through that, so when I see other people, I don't know what you might be going through. So when you're coming to me, saying something to me, a child acting out, I don't know what they're really feeling, and I don't know if they're free to tell me what they're feeling, but the only thing I can do is say, like you said I understand. I've calmed down. Let me talk to you. What could I possibly do? And if nothing else came out of that, that came out of it. We all come with something. And we don't know what it might be, but we can be kind." (Participant 2, FG2, Participant Themes 1, 2, and 3)

Model Construct:
Relational process: Adult learning techniques

Qualitative Themes:
Participant Theme 2: Experienced connection and validation during the course (9 speakers)
Participant Theme 3: Experienced healing from the course (9 speakers)
Trainer Theme 4: Trainers provided connection (8 speakers)

Example Quotes:
"We understood what each and everybody was going through. And there were some personal things that we kinda all related to, as well as professional, so we could relate to one another on different levels just from this alone. We didn't really know each other, we were all from different places, except for the person who you came with [if participating in the ETA course with a co-teacher], so just to know that, just from taking this class [ETA course], 'oh I'm not alone, other people are feeling like this, it's not just me, it's not just my [early childhood] classroom, it's not just my [early childhood] program.' So we all had a commonality and didn't even realize it until after we all sat and talked at this trauma awareness [course]. I just thought that what if we all did that, like what if we all could just talk

© 2019 Whitaker RC et al. *JAMA Network Open.*

like, across the board and we would all really see, wow, we're all going through a lot, maybe we can all understand each other more and maybe things will go better." (Participant 3, FG2, Participant Theme 2)

"And this [ETA] course helped me so much…my son passed away in 2016 and I didn't realize, my baby boy, thirty years old now, he came to me and he said, 'Mom since you took that course, you are so different.' I learned how to talk and handle him at home when I was just on him and beating [him] down. It's deep." (Participant 2, FG1, Participant Theme 3)

"I think in this class [ETA course], the hope also came from understanding themselves and what has happened to them, and having this space to share that and to say, 'Okay, this makes sense. Oh what I'm doing, why I'm doing it, understanding the brain, understanding trauma now.' I think that's the huge switch, and then they are able to say, 'Oh my gosh, this child's coming in, what has happened to them?'…we started the room with the brain break, not knowing where they're from…a lot of stories came [out] about themselves, which honestly like all of us, they may not have termed that as trauma, they may not have understood or made sense of it all, so when they understand that, they then are able to move through that in a space that's safe, and then give to others. I think that's very different than other trainings. You know, truly begin with yourself, and it may not look so pretty, and to have the tears, to be able to say, 'this is not what I thought I was gonna share, ugh' but then to release [it]. 'I feel good, oh I'm coming back to the next session,' you know what I mean? You're creating that space so then everyone's having the connection. When they have tears not to stop but to pass the tissues to them." (Trainer 2, FG3, Trainer Themes 1 and 4)

"I think that at a very basic level of empathy and compassion towards themselves and other human beings, being able to really realize that the dynamics that are at work in me and in the children that I work with, those same dynamics are in everybody. I do feel like from the first [session] to session six, that there is a sense of really, when someone else is talking over there, like wow I see our own shared humanity in them in the sense of heightened listening and respect and vulnerability. When the magic really happens, everybody is in tune with each other when one person's sharing, it's no longer just the trainers who are there, but it's really kind of this group mind thing going on." (Trainer 9, FG3, Trainer Themes 2 and 4)

© 2019 Whitaker RC et al. *JAMA Network Open.*

eTable 1. Detailed Instrument Descriptions for the Trial Outcome Measures

Construct	Instrument	Description	Cronbach's alpha at baseline
**Relationship quality**			
Teacher-children conflict	Conflict subscale of a modified version of the Student-Teacher Relationship Scale (STRS)[4]	The modified version of the STRS required the respondent to reflect on their classroom as a whole (i.e., items refer to "children"), rather than selecting a particular child in the class (i.e., items in the original version refer to "child"). The conflict subscale consisted of 8 items reflecting negative relationship quality (e.g., "The children and I always seem to be struggling with each other."). Respondents chose the best response to each item, on a 5-point Likert-type scale, ranging from (1) "definitely does not apply" to (5) "definitely applies." Responses were summed, with higher scores indicating greater conflict.	.82
Trust with adults			
Trust with parents	Modified version of the Trust in Work Relationships with Parents (Teacher-Parent Trust items) subscale of the Trust in Schools Instruments[5]	We replaced "school" with "early childhood program" and "teachers" with "staff." Trust in Work Relationships with Parents includes 13 items (e.g., "Parents have confidence in the expertise of the staff."). Response options for 9 items used 4-point Likert-type scales (either "strongly disagree" [1] to "strongly agree" [4] or "not at all" [1] to "to a great extent" [4]), and response options for 4 items used a 5-point Likert-type scale ("none" [1] to "nearly all" [5]). After reverse-coding 2 items, item scores were divided by 4 (for 4-point scales) and by 5 (for 5-point scales). A scale summary score (possible range 3.05 to 13.00) was then computed by adding scores for the 13 items, with higher summary scores reflecting greater trust with parents.	.86
Trust with other staff	Modified version of the Trust in Work Relationships with Other Staff (Teacher-Teacher Trust items) subscale of the Trust in Schools Instruments[5]	We replaced "school" with "early childhood program" and "teachers" with "staff." Trust in Work Relationships with Other Staff included 7 items (e.g., "To what extent do you feel respected by other staff?"). Response options for 6 items used 4-point Likert-type scales (either "strongly disagree" [1] to "strongly agree" [4] or "not at all" [1] to "to a great extent" [4]), and response options for 1 item used a 5-point Likert-type scale ("none" [1] to "nearly all" [5]). Item scores were divided by 4 (for 4-point scales) and by 5 (for 5-point scale). A scale summary score (possible range 1.70 to 7.00) was then computed by	.85

© 2019 Whitaker RC et al. *JAMA Network Open.*

Construct	Instrument	Description	Cronbach's alpha at baseline
		adding scores for the 7 items, with higher scores reflecting greater trust with other staff.	
Trust with supervisors	Modified version of the Trust in Work Relationships with Supervisors (Teacher-Principal Trust items) subscale of the Trust in Schools Instruments[5]	We replaced "school" with "early childhood program," "teachers" with "staff," and "principal" with "supervisor." Trust in Work Relationships with Supervisors included 9 items (e.g., "My supervisor has confidence in the expertise of his or her staff."). Response options used 4-point Likert-type scales (either "strongly disagree" [1] to "strongly agree" [4] or "not at all" [1] to "to a great extent" [4]). Item scores were divided by 4, and a scale summary score (possible range 2.25 to 9.00) was then computed by adding scores for the 9 items, with higher scores reflecting greater trust with supervisors.	.93
**Relational capacities**			
Emotion regulation			
Cognitive reappraisal	Cognitive Reappraisal subscale of the Emotion Regulation Questionnaire[6]	Cognitive Reappraisal included 6 items (e.g., "I control my emotions by *changing the way I think* about the situation I'm in."). Response options included a 7-point Likert-type scale that ranged from "strongly disagree" (1) to "strongly agree" (7). Scores were determined by calculating the average of the subscale item scores (range 1-7), with higher scores reflecting greater cognitive reappraisal (which was the hypothesized direction of the intervention impact).	.82
Expressive suppression	Expressive Suppression subscale of the Emotion Regulation Questionnaire[6]	Expressive Suppression included 4 items (e.g., "I control my emotions by *not expressing them*."). Response options included a 7-point Likert-type scale that ranged from "strongly disagree" (1) to "strongly agree" (7). Scores were determined by calculating the average of the subscale item scores (range 1-7), with lower scores reflecting less expressive suppression (which was the hypothesized direction of the intervention impact).	.66
Dispositional mindfulness	Cognitive and Affective Mindfulness Scale-Revised (CAMS-R)[7]	The 12-item CAMS-R was used as a self-report measure of the tendency or disposition to be mindful in daily life. In this single factor scale, each item describes an attitude or approach towards the experience of one's emotions or thoughts in four areas—focusing attention, being oriented to the present moment, being aware of an experience, and having an attitude of acceptance or nonjudgment towards an experience. Response options included a 4-point Likert-type scale that ranged from "rarely/not at all" (1) to "almost always" (4). A total score was	.83

© 2019 Whitaker RC et al. *JAMA Network Open.*

Construct	Instrument	Description	Cronbach's alpha at baseline
		determined by first reverse-coding items where necessary and then summing all item scores (range 12-48), with higher scores reflecting greater mindfulness.	
Empathy			
Perspective-taking	Perspective-Taking subscale of the Interpersonal Reactivity Index[8]	Perspective Taking included 7 items (e.g., "I sometimes find it difficult to see things from the 'other guy's' point of view."). Response options included a 5-point Likert-type scale that ranged from "this doesn't describe me well" (0) to "this describes me very well" (4). Scores[9] were determined by first reverse-coding items where necessary and then summing the subscale item scores (range 0-28), with higher scores indicating greater perspective-taking.	.65
Empathic concern	Empathic Concern subscale of the Interpersonal Reactivity Index[8]	Empathic Concern included 7 items (e.g., "When I see someone being taken advantage of, I feel kind of protective towards them."). Response options included a 5-point Likert-type scale that ranged from "this doesn't describe me well" (0) to "this describes me very well" (4). Scores[9] were determined by first reverse-coding items where necessary and then summing the subscale item scores (range 0-28), with higher scores indicating greater empathic concern.	.52
Personal distress	Personal Distress subscale of the Interpersonal Reactivity Index[8]	Personal Distress included 7 items (e.g., "I sometimes feel helpless when I am in the middle of a very emotional situation."). Response options included a 5-point Likert-type scale that ranged from "this doesn't describe me well" (0) to "this describes me very well" (4). Scores[9] were determined by first reverse-coding items where necessary and then summing the subscale item scores (range 0-28), with lower scores indicating less personal distress (which was the hypothesized direction of the intervention impact).	.74
Compassion satisfaction	Compassion Satisfaction subscale of the Professional Quality of Life Scale[10]	Compassion Satisfaction included 10 items assessing the positive aspects of being able to help others (e.g., "I believe I can make a difference through my work."). We instructed participants to think of their "helping" in terms of their job as an early childhood educator. Participants rated the frequency of each experience on a 5-point Likert-type scale that ranged from "never" (1) to "very often" (5). Scores were determined by summing the item scores and converting the raw score to a t-score, with higher scores reflecting greater compassion	.88

© 2019 Whitaker RC et al. *JAMA Network Open.*

Construct	Instrument	Description	Cronbach's alpha at baseline
		satisfaction (which was the hypothesized direction of the intervention impact).	
Attitudes about trauma-informed care	Attitudes Related to Trauma-Informed Care (ARTIC) Scale[11]	The 10-item ARTIC Scale was used to measure attitudes about trauma-informed care. In the directions for this instrument, we instructed participants that "the term 'client' in the following questions means 'families' served in Head Start." Response options included a 7-point scale with the participant selecting a response along the continuum between two options that "best represents your personal belief during the past 30 days at your job" (e.g., "If clients say or do disrespectful things to me, it makes me look like a fool in front of others." (1) to "If clients say or do disrespectful things to me, it doesn't reflect badly on me." (7)). A total score was determined by first reverse-coding items where necessary and then calculating the average score (range 1-7), with higher scores reflecting more favorable attitudes about trauma-informed care.	.82
**Health and well-being**			
Burnout			
Emotional exhaustion	Emotional Exhaustion subscale from the Maslach Burnout Inventory- Educators Survey[12]	Emotional Exhaustion included 9 items. Response options ranged from "never" (0) to "every day" (6) on a 7-point Likert-type scale. Scores were determined by calculating the average of the subscale items (range 0-6), with a higher average indicating more emotional exhaustion, reflecting greater burnout.	.91
Depersonalization	Depersonalization subscale from the Maslach Burnout Inventory- Educators Survey[12]	Depersonalization included 5 items. Response options ranged from "never" (0) to "every day" (6) on a 7-point Likert-type scale. Scores were determined by calculating the average of the subscale items (range 0-6), with a higher average indicating more depersonalization, reflecting greater burnout.	.63
Personal accomplishment	Personal Accomplishment subscale from the Maslach Burnout Inventory- Educators Survey[12]	Personal Accomplishment included 8 items. Response options ranged from "never" (0) to "every day" (6) on a 7-point Likert-type scale. Scores were determined by calculating the average of the subscale items (range 0-6), with a higher average indicating more personal accomplishment, reflecting less burnout.	.67
Secondary traumatic stress	Secondary Traumatic Stress subscale of the Professional Quality of Life Scale[10]	Secondary Traumatic Stress (a component of compassion fatigue) included 10 items assessing the secondary trauma felt by those working with people experiencing high levels of trauma (e.g., "I feel depressed because of the traumatic experiences of	.76

© 2019 Whitaker RC et al. *JAMA Network Open.*

Construct	Instrument	Description	Cronbach's alpha at baseline
		the people I help."). We instructed participants to think of their "helping" in terms of their job as an early childhood educator. Participants rated the frequency of each experience on a 5-point Likert-type scale that ranged from "never" (1) to "very often" (5). Scores were determined by summing the item scores and converting the raw score to a t-score, with lower scores reflecting less secondary traumatic stress (which was the hypothesized direction of the intervention impact).	
Job satisfaction	Modified question from the Gallup Well-Being Work Index	We assessed job satisfaction with the following item: "How satisfied are you with your work as an early childhood educator?" Response options ranged from "very dissatisfied" (1) to "very satisfied" (6).	n/a
Health-related quality of life			
Mentally unhealthy days	One question from the Healthy Days core questions from the CDC Behavioral Risk Factor Surveillance System[13]	One question asked about the number of days during the past 30 days when mental health ("which includes stress, depression, and problems with emotions") was not good (range 0-30 days). We developed a binary measure of any mentally unhealthy days (any mentally unhealthy days vs. no mentally unhealthy days).	n/a
Physically unhealthy days	One question from the Healthy Days core questions from the CDC Behavioral Risk Factor Surveillance System[13]	One question asked about the number of days during the past 30 days when physical health ("which includes physical illness and injury") was not good (range 0-30 days). We developed a binary measure of any physically unhealthy days (any physically unhealthy days vs. no physically unhealthy days).	n/a
Days poor health interferes	One question from the Healthy Days core questions from the CDC Behavioral Risk Factor Surveillance System[13]	One question asked, "During the past 30 days, for about how many days did poor physical or mental health keep you from doing your usual activities, such as self-care, work, or recreation?" (range 0-30 days). We developed a binary measure of any days poor health interferes (any days poor health interferes vs. no days poor health interferes).	n/a
Sleep			
Sleep duration	One question from the Pittsburgh Sleep Quality Index[14]	The sleep duration question asked "How many hours of actual sleep did you get at night during the past 30 days? (This may be different than the number of hours you spend in bed)." Response options included a range of 3-15 for hours (at 1-hour increments) and 0-45 for minutes (at 15-minute increments), with hours and minutes combined for sleep duration measured in hours. We	n/a

© 2019 Whitaker RC et al. *JAMA Network Open.*

Construct	Instrument	Description	Cronbach's alpha at baseline
		developed a binary measure of sleep duration (<7 hours/night vs. ≥7 hours/night).	
Quality	One question from the Pittsburgh Sleep Quality Index[14]	The sleep quality question asked "How would you rate your sleep quality overall during the past 30 days?" Response options included a 4-point Likert-type scale that ranged from "very bad" (1) to "very good" (4).	n/a

Note: Missing values at baseline: trust with parents (1 participant missing 1 item), compassion satisfaction (1 participant missing 1 item), depersonalization (2 participants each missing 1 item), and days poor health interferes (1 participant missing the item). For items with missing values, the missing value was replaced with the sample median value for that item. For the variable "days poor health interferes," the one participant missing this item was excluded from the analysis for this outcome.

© 2019 Whitaker RC et al. *JAMA Network Open.*

eTable 2. Neighborhood Characteristics of Eligible Early Childhood Education Sites, by Participation in the Trial

Neighborhood characteristic of site	All sites (n=133)	Site with classroom(s) participating in trial: Yes (n=38)	Site with classroom(s) participating in trial: No (n=95)
Percentage of population below federal poverty level, mean (SD)[a]	30.6 (27.0)	35.3 (33.5)	28.7 (23.8)
Extreme poverty neighborhood, No. (%)[b]	46 (34.6%)	16 (42.1%)	30 (31.6%)
Neighborhood percentile for CDC Social Vulnerability Index (SVI):			
Socioeconomic theme, mean (SD)[c]	76.4 (25.6)	77.5 (28.5)	76.0 (24.6)
Racial/ethnic composition[d]			
Percentage White, non-Hispanic, mean (SD)	29.4 (29.0)	23.5 (26.4)	31.8 (29.7)
Percentage Black, non-Hispanic, mean (SD)	44.0 (35.0)	50.0 (36.5)	41.6 (34.3)
Percentage Other race, non-Hispanic, mean (SD)	9.8 (10.8)	9.3 (11.5)	10.0 (10.6)
Percentage Hispanic, any race, mean (SD)	16.8 (22.7)	17.2 (24.9)	16.6 (21.9)
Violent crimes rate (per 1,000 residents per year), mean (SD)[e]	12.3 (7.9)	13.0 (7.6)	12.0 (8.0)

Note: CDC, Centers for Disease Control and Prevention. Neighborhood defined as the census tract in which the early childhood education site was located.

[a] Percentage of population (all families with related children of householder under 5 years of age) below poverty level was derived from the U.S. Census Bureau, 2011-2015 American Community Survey (ACS) 5-Year Estimates (data available at: https://factfinder.census.gov/faces/nav/jsf/pages/download_center.xhtml).

[b] Extreme poverty neighborhood is defined as a neighborhood (census tract) in which ≥40% of households with children under 5 years of age live below the federal poverty level.

[c] The CDC SVI (2016): Socioeconomic theme is a percentile that represents the relative vulnerability of every U.S. Census tract with respect to socioeconomic status (factors include below poverty, unemployed, income, and no high school diploma). Higher values indicate greater vulnerability. One site (not in the trial) was located in a census tract for which SVI data were not reported by the CDC (data available at: https://svi.cdc.gov/data-and-tools-download.html).

[d] Racial/ethnic composition was derived from the U.S. Census Bureau, 2011-2015 ACS 5-Year Estimates.

[e] Violent crime was derived from the count of violent crime incidents (homicide, rape, robbery, aggravated assault) in 2015 in each census tract as obtained from the City of Philadelphia (data available at: http://metadata.phila.gov/#home/datasetdetails/5543868920583086178c4f8e/). Census tract population data obtained from the U.S. Census Bureau, 2011-2015 ACS 5-Year Estimates.

© 2019 Whitaker RC et al. *JAMA Network Open.*

eTable 3. Subgroup Comparisons of the Effect of Enhancing Trauma Awareness on Teacher-Children Conflict Scores

Subgroups based on teacher characteristics	Baseline mean (SD)		Follow-up mean (SD)		Mean difference at follow-up		p[b]
	Intervention	Control	Intervention	Control	Unadjusted (95% CI)	Adjusted[a] (95% CI)	
In sites with intervention and control classrooms[c]							
Yes (n=37)	14.4 (3.3)	16.1 (6.5)	15.1 (5.7)	15.1 (3.3)	-0.05 (-3.03, 2.94)	0.5 (-2.1, 3.2)	.89
No (n=56)	16.3 (5.6)	15.7 (5.4)	16.0 (5.0)	15.4 (4.2)	0.6 (-1.9, 3.2)	0.6 (-1.4, 2.6)	
Lead teacher							
Yes (n=45)	16.6 (6.0)	16.9 (6.5)	15.4 (5.5)	15.6 (3.2)	-0.1 (-2.8, 2.5)	-0.05 (-2.1, 2.0)	.43
No (n=48)	15.0 (4.3)	15.0 (5.2)	16.0 (4.9)	14.9 (4.2)	1.1 (-1.5, 3.8)	1.1 (-0.9, 3.1)	
>15 years ECE experience[d]							
Yes (n=43)	15.5 (4.7)	14.1 (4.6)	15.3 (4.4)	14.3 (4.3)	0.9 (-1.7, 3.6)	0.5 (-1.7, 2.8)	.95
No (n=48)	15.7 (5.6)	17.4 (6.5)	16.0 (6.0)	16.0 (3.0)	-0.04 (-2.76, 2.68)	0.6 (-1.5, 2.8)	
Baseline ACE score ≥3							
Yes (n=34)	15.8 (4.2)	15.4 (4.9)	16.5 (4.4)	15.2 (4.1)	1.3 (-1.7, 4.3)	1.0 (-1.0, 3.0)	.61
No (n=59)	15.8 (5.7)	16.2 (6.4)	15.3 (5.6)	15.3 (3.5)	0.01 (-2.42, 2.44)	0.3 (-1.8, 2.3)	
Teaching in a Head Start classroom							
Yes (n=62)	16.3 (4.7)	16.0 (6.2)	17.0 (5.8)	15.2 (3.8)	1.8 (-0.6, 4.2)	1.8 (-0.3, 3.8)	.16
No (n=31)	15.2 (5.6)	15.7 (4.6)	14.3 (4.0)	15.2 (3.5)	-0.9 (-4.1, 2.2)	-0.6 (-3.0, 1.8)	
Bachelor's degree or higher							
Yes (n=47)	15.8 (5.5)	15.9 (4.6)	14.5 (4.5)	15.9 (3.7)	-1.4 (-3.8, 1.0)	-1.3 (-3.2, 0.5)	**.01**
No (n=46)	15.7 (4.9)	15.9 (7.2)	16.9 (5.6)	14.5 (3.7)	2.4 (-0.4, 5.2)	2.5 (0.4, 4.6)	

Note: ACE, adverse childhood experiences; ECE, early childhood education.

[a] Adjusted for baseline values of teacher-children conflict scores. Differences = intervention minus control.

[b] *P* value for the interaction term of subgroup variable (Yes[1]/No[0]) X study group variable (Intervention[1]/Control[0]) in a multi-level (classroom and site levels) regression model predicting follow-up teacher-children conflict scores adjusting for baseline values of teacher-children conflict scores. Boldface values indicate statistical significance at the p<.05 level for the interaction term in the regression model.

[c] In the analytic sample (n= 93), there were 8 sites (37 teachers, 24 control and 13 intervention) in which there were classrooms assigned to both intervention and controls arms and 29 sites (56 teachers, 22 control and 34 intervention) in which all classrooms were assigned to either an intervention or control group (18 sites had multiple classrooms but each of these sites had classrooms assigned to the same condition).

[d] Two participants were missing data on the variable ECE experience, n= 91. For all other subgroup comparisons, n= 93.

© 2019 Whitaker RC et al. *JAMA Network Open.*

eTable 4. Effect of Enhancing Trauma Awareness at Delayed Follow-up on Measures of Relationship Quality, Relational Capacities, and Health and Well-being[a]

Variable and hypothesized direction of change	Baseline		Delayed follow-up		Mean or prevalence difference at delayed follow-up (Intervention vs Control)			Magnitude of effect (95% CI)[d]
	Intervention (n= 47)	Control (n=46)	Intervention (n= 47)	Control (n=46)	Unadjusted (95% CI)	Adjusted (95% CI)[b]	*p*[c]	
**Relationship quality**								
Decreased teacher-children conflict, mean (SD)	15.8 (5.1)	15.9 (5.9)	15.6 (4.4)	15.1 (4.8)	0.5 (-1.3, 2.4)	0.7 (-1.1, 2.4)	.45	0.15 (-0.24, 0.54)
Trust with adults								
Increased trust with parents, mean (SD)	10.3 (1.4)	10.2 (1.2)	10.1 (1.6)	9.8 (1.3)	0.2 (-0.4, 0.8)	0.02 (-0.55, 0.58)	.95	0.01 (-0.37, 0.40)
Increased trust with other staff, mean (SD)	5.4 (0.9)	5.3 (0.9)	5.5 (1.0)	5.2 (0.8)	0.2 (-0.1, 0.6)	0.2 (-0.1, 0.5)	.24	0.21 (-0.14, 0.55)
Increased trust with supervisors, mean (SD)	7.2 (1.3)	7.1 (1.4)	7.2 (1.3)	7.1 (1.7)	0.1 (-0.5, 0.7)	0.04 (-0.51, 0.59)	.89	0.02 (-0.34, 0.39)
**Relational capacities**								
Emotion regulation								
Increased cognitive reappraisal, mean (SD)	5.3 (1.2)	5.1 (1.0)	5.3 (1.3)	5.0 (1.2)	0.3 (-0.2, 0.8)	0.2 (-0.3, 0.6)	.45	0.13 (-0.21, 0.47)
Decreased expressive suppression, mean (SD)	3.3 (1.1)	3.4 (1.3)	3.4 (1.3)	3.3 (1.2)	0.1 (-0.5, 0.6)	0.1 (-0.3, 0.5)	.69	0.07 (-0.28, 0.43)
Increased dispositional mindfulness, mean (SD)	38.0 (5.8)	37.0 (5.8)	37.4 (5.3)	35.6 (5.1)	1.8 (-0.4, 3.9)	0.9 (-0.8, 2.7)	.30	0.18 (-0.16, 0.52)
Empathy								
Increased perspective-taking, mean (SD)	21.3 (3.8)	20.7 (4.0)	21.0 (4.3)	20.3 (4.6)	0.7 (-1.2, 2.5)	0.3 (-1.2, 1.8)	.69	0.07 (-0.28, 0.42)
Increased empathic concern, mean (SD)	23.3 (3.0)	22.0 (3.7)	22.6 (4.3)	22.0 (4.1)	0.7 (-1.1, 2.4)	-0.2 (-1.7, 1.3)	.80	-0.05 (-0.40, 0.31)
Decreased personal distress, mean (SD)	8.8 (4.7)	8.8 (5.2)	8.5 (4.3)	8.8 (4.1)	-0.3 (-2.0, 1.4)	-0.3 (-1.7, 1.0)	.64	-0.08 (-0.41, 0.25)
Increased compassion satisfaction, mean (SD)	49.0 (11.0)	50.9 (9.2)	49.8 (10.4)	50.4 (10.0)	-0.6 (-4.8, 3.6)	-0.1 (-3.4, 3.1)	.93	-0.01 (-0.34, 0.31)
Increased ARTIC, mean (SD)	5.6 (1.0)	5.5 (0.9)	5.4 (1.1)	5.4 (1.0)	0.01 (-0.42, 0.44)	-0.04 (-0.39, 0.31)	.83	-0.04 (-0.38, 0.30)
**Health and well-being**								
Burnout								
Decreased emotional exhaustion, mean (SD)	1.8 (1.2)	1.9 (1.4)	1.8 (1.2)	1.8 (1.2)	0.01 (-0.51, 0.52)	0.03 (-0.37, 0.42)	.90	0.02 (-0.30, 0.34)
Decreased depersonalization, mean (SD)	0.5 (0.7)	0.7 (1.0)	0.5 (0.6)	0.7 (0.9)	-0.2 (-0.5, 0.1)	-0.1 (-0.4, 0.1)	.37	-0.15 (-0.48, 0.18)
Increased personal accomplishment, mean (SD)	5.2 (0.7)	5.0 (0.8)	5.0 (1.1)	5.0 (0.8)	-0.02 (-0.41, 0.38)	-0.1 (-0.4, 0.2)	.61	-0.09 (-0.46, 0.27)
Decreased secondary traumatic stress, mean (SD)	51.2 (10.0)	48.8 (10.4)	51.0 (10.7)	48.6 (9.4)	2.3 (-1.8, 6.5)	1.8 (-1.9, 5.5)	.34	0.18 (-0.19, 0.55)
Increased job satisfaction, mean (SD)	5.2 (1.1)	5.5 (0.6)	5.1 (1.2)	5.0 (1.4)	0.1 (-0.4, 0.7)	0.3 (-0.2, 0.8)	.29	0.21 (-0.18, 0.59)
Health-related quality of life								
Decrease in any mentally unhealthy days, No. (%)	28 (60.9%)	24 (52.2%)	30 (65.2%)	38 (82.6%)	-17.4 (-35.0, 0.2)	-20.3 (-38.9, -1.7)	.06	0.30 (0.08, 1.08)
Decrease in any physically unhealthy days, No. (%)	22 (46.8%)	23 (50.0%)	27 (57.4%)	31 (67.4%)	-9.9 (-29.5, 9.6)	-7.7 (-29.8, 14.4)	.49	0.68 (0.22, 2.05)
Decrease in any days poor health interferes, No. (%)	11 (23.4%)	11 (24.4%)	20 (42.5%)	24 (53.3%)	-10.8 (31.1, 9.5)	-11.7 (-36.2, 12.8)	.36	0.58 (0.18, 1.83)
Sleep								
Increase in sleep duration to ≥7 hrs./night, No. (%)	21 (45.6%)	17 (37.8%)	28 (60.9%)	19 (42.2%)	18.6 (-1.5, 38.8)	16.8 (-3.8, 37.5)	.12	2.22 (0.81, 6.10)
Increased quality, mean (SD)	2.9 (0.7)	2.8 (0.6)	3.0 (0.7)	2.9 (0.6)	0.1 (-0.1, 0.4)	0.1 (-0.2, 0.3)	.62	0.09 (-0.26, 0.44)

Note: ARTIC, attitudes related to trauma-informed care.

© 2019 Whitaker RC et al. *JAMA Network Open.*

[a] Of the 93 participants who had survey data for the primary outcome analysis (manuscript Table 2), 89 had data from a delayed follow-up survey administered 5 months after the Enhancing Trauma Awareness (ETA) course (intervention). The analysis was based on 93 participants. In the analysis, missing data for the 4 participants who did not complete the delayed follow-up survey were replaced with data from the immediate follow-up survey. At the time of this delayed follow-up survey, the waitlist control group had just completed the Enhancing Trauma Awareness (ETA) course. Therefore, the intervention-control comparisons shown here compare follow-up outcomes in those who had completed the ETA course 5 months earlier (original intervention arm) and those recently completing the ETA course (original control arm).

[b] Adjusted mean differences for continuous outcomes control for baseline level of the outcome variable and account for clustering of teacher outcomes at classroom and site levels. Adjusted prevalence differences for binary outcomes (mentally unhealthy days, physically unhealthy days, days poor health interferes, and sleep duration) were determined from predictive probabilities using only the fixed portion of the model.

[c] The *p* value is associated with the regression coefficient for the study group variable (Intervention[1]/Control[0]) in each linear multi-level (classroom and site levels) regression model (continuous outcomes) and with the odds ratio in each logistic multi-level (classroom and site levels) regression model (binary outcomes).

[d] The magnitude of the effect refers to an effect size. Effect size measures (adjusted for baseline values and clustering) were derived in linear models from the standardized partial coefficient for the binary study group variable and in logistic models from the odds ratio for that binary variable (mentally unhealthy days, physically unhealthy days, days poor health interferes, and sleep duration).

© 2019 Whitaker RC et al. *JAMA Network Open.*

eTable 5. Changes in Outcome Measures Between Baseline and Delayed Follow-up for Those in the Intervention Group Who Did and Did Not Participate in Focus Groups[a]

Variable and hypothesized direction of change	Baseline (Fall 2017)		Follow-up (Spring 2018)		Change (95% CI) in mean or prevalence (follow-up minus baseline)[b]		Difference[c] in change (95% CI) in mean or prevalence
	In focus group	Not in focus group	In focus group	Not in focus group	In focus group	Not in focus group	
**Relationship quality**							
Decreased teacher-children conflict, mean (SD)	14.8 (6.0)	16.2 (4.7)	14.7 (4.0)	16.0 (4.5)	-0.1 (-2.8, 2.7)	-0.2 (-2.1, 1.7)	0.1 (-3.2, 3.5)
Trust with adults							
Increased trust with parents, mean (SD)	10.6 (1.5)	10.2 (1.3)	10.3 (1.6)	10.0 (1.6)	-0.4 (-1.2, 0.4)	-0.2 (-0.8, 0.3)	-0.1 (-1.1, 0.8)
Increased trust with other staff, mean (SD)	5.4 (1.0)	5.4 (0.9)	5.8 (1.0)	5.3 (1.0)	0.43 (-0.03, 0.89)	-0.1 (-0.4, 0.2)	0.54 (-0.03, 1.10)
Increased trust with supervisors, mean (SD)	7.5 (1.3)	7.0 (1.3)	7.6 (1.4)	7.0 (1.3)	0.03 (-0.69, 0.76)	-0.02 (-0.52, 0.49)	0.05 (-0.83, 0.93)
**Relational capacities**							
Emotion regulation							
Increased cognitive reappraisal, mean (SD)	5.3 (1.4)	5.3 (1.2)	5.2 (1.4)	5.4 (1.1)	-0.2 (-0.8, 0.4)	0.1 (-0.3, 0.5)	-0.3 (-1.0, 0.4)
Decreased expressive suppression, mean (SD)	3.4 (0.9)	3.3 (1.2)	3.2 (1.2)	3.5 (1.4)	-0.1 (-0.8, 0.5)	0.1 (-0.3, 0.6)	-0.3 (-1.1, 0.5)
Increased dispositional mindfulness, mean (SD)	37.6 (5.8)	38.2 (5.8)	36.5 (5.6)	37.8 (5.2)	-1.1 (-3.4, 1.2)	-0.5 (-2.0, 1.1)	-0.6 (-3.4, 2.2)
Empathy							
Increased perspective-taking, mean (SD)	23.0 (3.8)	20.5 (3.6)	20.6 (4.9)	21.1 (4.0)	-2.4 (-4.7, -0.1)	0.6 (-1.0, 2.2)	-3.0 (-5.8, -0.2)
Increased empathic concern, mean (SD)	23.4 (3.8)	23.3 (2.6)	21.4 (4.7)	23.2 (4.0)	-2.0 (-4.1, 0.1)	-0.1 (-1.5, 1.4)	-1.9 (-4.5, 0.7)
Decreased personal distress, mean (SD)	7.5 (4.5)	9.4 (4.7)	8.1 (4.1)	8.7 (4.3)	0.6 (-1.6, 2.8)	-0.7 (-2.2, 0.8)	1.3 (-1.3, 3.9)
Increased compassion satisfaction, mean (SD)	46.5 (14.7)	50.1 (8.8)	46.6 (13.2)	51.4 (8.6)	0.1 (-4.1, 4.4)	1.3 (-1.7, 4.2)	-1.1 (-6.3, 4.0)
Increased ARTIC, mean (SD)	5.9 (1.0)	5.4 (1.0)	5.7 (1.7)	5.2 (1.0)	-0.2 (-0.6, 0.2)	-0.2 (-0.5, 0.1)	-0.01 (-0.56, 0.54)
**Health and well-being**							
Burnout							
Decreased emotional exhaustion, mean (SD)	1.7 (1.5)	1.9 (1.1)	1.7 (1.2)	1.9 (1.2)	0.01 (-0.63, 0.66)	-0.01 (-0.46, 0.43)	0.03 (-0.75, 0.81)
Decreased depersonalization, mean (SD)	0.6 (0.9)	0.5 (0.6)	0.4 (0.4)	0.6 (0.6)	-0.2 (-0.6, 0.2)	0.2 (-0.1, 0.4)	-0.4 (-0.8, 0.1)
Increased personal accomplishment, mean (SD)	5.1 (0.8)	5.2 (0.7)	4.9 (1.4)	5.0 (0.8)	-0.2 (-0.7, 0.4)	-0.2 (-0.5, 0.2)	0.01 (-0.63, 0.65)
Decreased secondary traumatic stress, mean (SD)	51.7 (13.2)	51.0 (8.2)	49.6 (11.5)	51.6 (10.4)	-2.1 (-7.1, 2.9)	0.6 (-2.9, 4.1)	-2.7 (-8.9, 3.4)
Increased job satisfaction, mean (SD)	5.3 (1.1)	5.2 (1.0)	5.1 (0.7)	5.1 (1.4)	-0.2 (-0.8, 0.4)	-0.03 (-0.47, 0.40)	-0.2 (-0.9, 0.6)
Health-related quality of life							
Decrease in any mentally unhealthy days, No. (%)	6 (42.9%)	22 (68.7%)	11 (78.6%)	19 (59.4%)	35.7 (2.0, 69.4)	-9.4 (-32.8, 14.0)	45.0 (18.9, 71.1)
Decrease in any physically unhealthy days, No. (%)	6 (40.0%)	16 (50.0%)	5 (33.3%)	22 (68.7%)	-6.7 (-41.1, 27.7)	18.7 (-4.9, 42.4)	-25.4 (-40.5, -10.3)
Decrease in any days poor health interferes, No. (%)	1 (6.7%)	10 (31.2%)	4 (26.7%)	16 (50.0%)	20.0 (-5.7, 45.7)	18.8 (-4.9, 42.4)	1.2 (-23.7, 26.1)
Sleep							
Increase in sleep duration to ≥7 hrs./night, No. (%)	9 (60.0%)	12 (38.7%)	9 (60.0%)	19 (61.3%)	0.0 (-0.3, 0.3)	22.6 (-1.7, 46.8)	-22.6 (-37.1, -8.1)
Increased quality, mean (SD)	3.1 (0.6)	2.8 (0.7)	3.2 (0.6)	2.9 (0.7)	0.1 (-0.2, 0.4)	0.1 (-0.1, 0.3)	-0.03 (-0.41, 0.35)

© 2019 Whitaker RC et al. *JAMA Network Open.*

Note: ARTIC, attitudes related to trauma-informed care.

[a] Sample limited to 47 of 48 subjects in the intervention group (15 of 15 in the focus group and 32 of 33 not the in focus group), who completed delayed follow-up surveys in spring 2018. Results were not analyzed using a multilevel model due to small numbers of units at the classroom and site level.

[b] For each continuous outcome, a change in the outcome (follow-up minus baseline) was first determined. Next, a linear regression model was used with change in the outcome as the dependent variable and focus group (In focus group [1]/Not in focus group [0]) as the independent variable. Using Stata (version 13.1) *margins* command, we determined the mean change (95% CI) for those in and not in the focus group. For each binary outcome (conducted separately for those in and not in the focus group), we used the Stata *prtest* command to calculate the differences (95% CI) of the prevalence at baseline and follow-up (follow-up minus baseline).

[c] Change for those in the focus group minus the change for those not in focus group. For each continuous outcome, a linear regression model was used with change in outcome as the dependent variable and focus group (In focus group [1]/Not in focus group [0]) as the independent variable. The regression coefficient (95% CI) for the focus group variable was used to determine the difference in the mean change (95% CI) between those in vs. not in the focus group. For each binary outcome, the difference in change in prevalence was determined using Stata's *prtesti* command. This command was used to test the equality of the mean change in prevalence between those in the focus group vs. those not in the focus group and to calculate the difference (95% CI) in these changes in prevalence (change in prevalence for those in the focus group minus change in prevalence for those not in focus group).

© 2019 Whitaker RC et al. *JAMA Network Open.*

eTable 6: Additional Teacher Focus Group Quotes Supporting Outcomes in the Conceptual Framework

Model Construct	Qualitative Theme	Example Quote
**Relationship quality**		
Teacher-children conflict	Theme 9: More mindful of trauma in managing the classroom (14 speakers)[a]<br><br>Theme 11: Created a more emotionally safe classroom (13 speakers)	"…and you would just let yourself maybe get, not, I don't want to say out of control, but just take yourself higher than you should, as far as getting worked up, and now you don't have [to], you can tell that, if you keep calm it brings the child down, so that's the way you should deal with it, and that's what I learned." (Participant 8, FG 1, Themes 9 and 11)<br><br>"Just this year, I have been told [by a child] 'Get out of my face, I don't have to listen to you, you're not my mother,' kicked at me, kicked and screamed. So what am I [to] do to try to bring this child down? And you know it's funny how you can. I am equipped to do that just from taking this course. When the child says that, don't go up here. You know, rage fire. Don't go up here." (Participant 6, FG 1, Themes 9 and 11)<br><br>"…how can I help kids? And the big thing is you have to help yourself first. And like I said, that made me start thinking about myself and when you start thinking about yourself and what fears and stuff you have, makes you calmer. You present a calmer front to children, and your attitude, like when they [the trainers] came in and they did the calmness, it calmed everybody else down. So that kind of flows over to the classroom. If you come in, and you start your day, you're not hyped, you get in and you're like, they could be all over the place and you go, 'oh alright, come over here honey,' and when somebody talks to you like that, it just gives you that feeling of calming and it sets the tone. So even the manner in which the trauma [class] was done, and going to that class gave you that sense of understanding that you can get everyone to buy into it…because you set the tone, and if you're in there and you're like a hot mess, 'Oh my god, hey, what's going on? Oh sit down.' That's how your class is gonna be." (Participant 2, FG2, Themes 9, 10, and 11)
Trust with parents	Theme 8: More sensitive to behaviors reflecting responses to trauma (15 speakers)	"I was just going to reflect on what some of y'all [others in the focus group] were saying as far as, being sensitive to the child, and it's like a circle, because it starts with us as teachers in the classroom, assistants and everybody that's with the child of course, and then with the parent. We know that children are coming from different types of environments and backgrounds and different things like that, so when we see a parent, reflected down to the child, and the child is bringing it to the classroom, we just felt we had to help her also, help the parent." (Participant 7, FG1, Themes 5 and 8)

© 2019 Whitaker RC et al. *JAMA Network Open.*

Model Construct	Qualitative Theme	Example Quote
		"…the parents…they just don't know like we didn't know. Informing them, getting them in the loop is the best thing. And how do we do that? …since we took the course, I've been sending them links, I've been taking pictures of paragraphs, I've been really informing them, sending them videos. Whether they see it or not, I just keep sending it to them until I bother them, until they're like 'Ugh, what is this [teacher] doing to me?' so they open it and read it or they watch the video. So I've been sending them a lot….having informal or formal meetings with them really helps them because they can see that we care about their children, and they see that differently. At the beginning of the school year they just drop their children because it's a school. But they don't see it as a school now. They see it differently. They see it as people that love my child so I better take my child to school. It means a lot." (Participant 1, FG2, Themes 8 and 11)
**Relational capacities**		
Empathy	Theme 5: Greater understanding of children's experiences as traumatic (10 speakers)<br><br>Theme 7: Greater recognition of trauma in non-work relationships (10 speakers)<br><br>Theme 8: More sensitive to behaviors reflecting responses to trauma (15 speakers)<br><br>Theme 9: More mindful of trauma in managing the classroom (14 speakers)	"That's what the class helped me do. Instead of seeing them just as the children, sometimes I see me in them. I see myself. And what they're going through and I've been there, I understand." (Participant 3, FG1, Themes 5 and 8)<br><br>"And so you think about us as adults, what are these children seeing, and then some of the things we hear coming from the children, and they're three, four, and five [years old], and their experience of some of these things, looking at these questions [about adverse childhood experiences] that we didn't experience anything like that in my family…so this child is coming to my classroom, yeah I think it makes you more sensitive. This class made me more sensitive, just looking at the questions [about adverse childhood experiences] because I never was asked these questions before, and some things these children are dealing with, these issues, me as an adult never did, made me be more sensitive to them because they feel some of this or they see it, but they think this is normal. And it's really not and you hear about this sort [of thing] on the news, so it makes you just become more aware of their needs and because on the whole, I'm a black mom, not taking any offense to anybody's color, but my mom was the type…she was firm. So I'm a firm mom as well. These kids, they're getting just the opposite of what I got, so I had to be more aware, more sensitive to them…we see some of the people bringing them to school and dropping them off, and it's like, what are they dealing with in the home? I became more sensitive to them because, what are you going home to? You know, it's something." (Participant 6, FG1, Themes 5 and 8)

© 2019 Whitaker RC et al. *JAMA Network Open.*

Model Construct	Qualitative Theme	Example Quote
		"…when you think about an infant not being cared for and not really being able to make the connections, it's heartbreaking because will they ever be able to connect because this [trauma] was so early on and one thing I have taken out of my vocabulary is 'you should know better.' You don't know whether they [children] know better or not. You just have to work with what you have and teach them from that point because you don't know what they know and what they've been through, because like I've said, early on the connections, you knew you're supposed to talk to babies and do things but to have a child that's really broken so early on is trauma within itself even after they get into a better situation. It's a scary thought, and you wonder why people walk around the way they do, but you don't know, the first year of their life may have been trauma, and then later on [we] say, well why can't they connect? Sometimes I think it's so deep and so early, it's scary." (Participant 2, FG2, Themes 5 and 8)<br><br>"I think so often, it's like calm down, or stop crying, or you know, you don't have to act, and the idea…of helping a child, or I feel an individual, because sometimes it could be the parent, too. It's okay to have these feelings or I understand these feelings and making that connection first, because we don't know what's happening or what's going on, and we can't tell someone else how to feel or tell them to come out of it, and how important that is, making that connection, and letting another human being know, 'it's okay' or 'I understand' and then maybe you can move on from there, meeting them where they're at." (Participant 4, FG2, Themes 8 and 9)
Emotion regulation	Theme 3: Experienced healing from the course (9 speakers)<br><br>Theme 9: More mindful of trauma in managing the classroom (14 speakers)<br><br>Theme 10: Used self-care strategies to remain calm (9 speakers)	"I think I just try to take care of myself more. I think [there were] like one or two pages [in the ETA course readings] of different things you could do to try to help calm yourself down. Take a bath. And you know what? I have teenagers, and they drive me crazy, and I'm always doing stuff for them, them, them. So even if I can't take myself on a lavish cruise or a trip or something, just do little things to take care of me, so I can stay focused, so I can stay calm, so I can try to stay stress free, not just in my own personal life, but at work as well. So I think that was also one of the big things that I took from [the ETA course], to make sure that I take care of myself." (Participant 3, FG2, Theme 10)<br><br>"And I think it's like you're saying, it's a healing process. The tissue [box] passing is, you're speaking about what you're feeling and that's part of healing, speaking about what you're feeling. And so you're enthusiastic about it because it's coming out, and you're feeling good about it. And that's what the class did." (Participant 8, FG1, Theme 3)

© 2019 Whitaker RC et al. *JAMA Network Open.*

Model Construct	Qualitative Theme	Example Quote
Dispositional mindfulness	Theme 9: More mindful of trauma in managing the classroom (14 speakers)	"And to really get a chance to understand what's going on with these children, why their behavior is sometimes out of line, and why they react the way they do. So we were just pre-judging them; just step back and think, something is going on, and we need to find out, maybe just back off a little bit and handle them differently, and sometimes it works and sometimes it doesn't, but at least it gave us an idea about how to think about it." (Participant 4, FG1, Themes 5, 8, and 9)<br><br>"Instead of going the same way I would normally, I would go a different route, and it helped. I had one child in particular, [who needed] to be evaluated….Well the ladies [evaluators] came in [to the classroom]. When somebody came into the room, he [child being evaluated] knew, something's going on. The boys had their coats on so instantly I was like think, think, think, and I remembered this class, and I said, slow down, tell your partner take the girls, leave the boys, and when I did, we left the boys, 'boys just take your coats off for a little bit.' So the ladies are waiting, 'take your coats off boys, we're just going to stay here for a little bit, and we are gonna sing for a while.' So gradually, and it took about ten to fifteen minutes, and I eased him [child being evaluated] over to the observers, and once he finally became comfortable, and they had gadgets, and he was involved, I said, 'boys put your coats on quietly and we'll take you outside' and someone came and took them out, and everything was smooth. Now if I had done it the other way [referring to her earlier statement of 'Come on, come on, you have to go, we have to go, come on, just grab my hand'], he would have been like this and not understanding, but I just had to…think about what I was doing. He got to play, you know, when he thought he was playing with the observers, and I didn't have to go from him crying, and the tantrum because he is the type that would get on the floor and just like throw his head." (Participant 6, FG1, Themes 9 and 11)
Attitudes about trauma-informed care	Theme 5: Greater understanding of children's experiences as traumatic (10 speakers)<br><br>Theme 8: More sensitive to behaviors reflecting responses to trauma (15 speakers)	"For me, it [the iceberg analogy presented in the ETA course to suggest that emotions arising from past experiences may be occurring below the surface of an individual's outward behavior] was just learning about the child and what it meant was trying to get inside, and learn more about them, and build more of a connection. Yeah for me the iceberg was the wall, you know. And just trying to get to know them better, and make the connection, and build a bond with them, and that was the iceberg for me, to have it melt a bit, that's how I've looked at it." (Participant 1, FG1, Theme 8)<br><br>"Because some of the parents are rough, they're traumatic. And so I say, well we have to show them something different, because the environment that they're in,

© 2019 Whitaker RC et al. *JAMA Network Open.*

Model Construct	Qualitative Theme	Example Quote
		they're not receiving the love, the compassion that we give them here, and the parents will tell you, well I don't know how you get them to act that way. Because we're doing something different. It [the ETA course] affected [my working relationship with my co-teacher]. Before the course, you didn't know what words to put on what you were doing, but after the course we're like, see we went there, we learned this, and this is how we do this, while we've been doing it all the time, now we can label it as [such]. And we even encouraged the director, we've learned this in this class [ETA course], we have to change because of the environment we're working in and the demographics. When these children come in, they need us, they're going through trauma, a lot of our children are in shelters, things like that, 'Oh Miss [teacher's name], we don't have a book at our house, or we don't have a house.'" (Participant 5, FG1, Themes 5, 9, and 11)

Note: FG, focus group.

[a] Indicates the number of focus group participants (out of 15) who had at least one comment during the focus group that supported the theme.

© 2019 Whitaker RC et al. *JAMA Network Open.*

eReferences

1. Wagenhals LD. *ETA: Enhancing Trauma Awareness*. North Wales, PA: Lakeside Global Institute; 2017.
2. Rosenbloom D, Williams MB, Watkins BE. *Life After Trauma: A Workbook for Healing*. 2nd ed. New York, NY: Guilford Press; 2010.
3. Bloom SL. *Creating Sanctuary: Toward the Evolution of Sane Societies*. Revised ed. New York, NY: Routledge; 2013.
4. Whitaker RC, Dearth-Wesley T, Gooze RA. Workplace stress and the quality of teacher–children relationships in Head Start. *Early Child Res Q*. 2015;30:57-69. doi:10.1016/j.ecresq.2014.08.008. Available at: https://curry.virginia.edu/uploads/resourceLibrary/STRS-SF_modification_for_staff_wellness_survey.pdf.
5. Bryk AS, Schneider BL. *Trust in Schools: A Core Resource for Improvement*. New York, NY: Russell Sage Foundation; 2002.
6. Gross JJ, John OP. Individual differences in two emotion regulation processes: implications for affect, relationships, and well-being. *J Pers Soc Psychol*. 2003;85(2):348-362. doi:10.1037/0022-3514.85.2.348
7. Feldman G, Hayes A, Kumar S, Greeson J, Laurenceau JP. Mindfulness and emotion regulation: the development and initial validation of the Cognitive and Affective Mindfulness Scale-Revised (CAMS-R). *J Psychopathol Behav Assess*. 2007;29(3):177-190. doi:10.1007/s10862-006-9035-8
8. Davis MH. A multidimensional approach to individual differences in empathy. *JSAS Catalog of Selected Documents in Psychology*. 1980;10:85.
9. Pulos S, Elison J, Lennon R. The hierarchical structure of the Interpersonal Reactivity Index. *Soc Behav Pers*. 2004;32(4):355-359.
10. Stamm BH. Professional Quality of Life: compassion satisfaction and fatigue, version 5 (ProQOL). https://proqol.org/uploads/ProQOL_5_English.pdf. Published 2009. Accessed January 16, 2019.
11. Baker CN, Brown SM, Wilcox PD, Overstreet S, Arora P. Development and psychometric evaluation of the Attitudes Related to Trauma-Informed Care (ARTIC) scale. *School Ment Health*. 2016;8(1):61-76. doi:10.1007/s12310-015-9161-0
12. Maslach C, Jackson SE, Schwab RL. Maslach Burnout Inventory- Educators Survey (MBI-ES). In: Maslach C, Jackson SE, Leiter MP, ed. *Maslach Burnout Inventory Manual*. 3rd ed. Palo Alto, CA: Consulting Psychologists Press; 1996. Available at: https://www.mindgarden.com/316-mbi-educators-survey.
13. Centers for Disease Control and Prevention. Health-related quality of life (HRQOL). https://www.cdc.gov/hrqol/index.htm. Updated October 31, 2018. Accessed January 16, 2018.
14. Buysse DJ, Reynolds CF, Monk TH, Berman SR, Kupfer DJ. The Pittsburgh Sleep Quality Index: a new instrument for psychiatric practice and research. *Psychiatry Res*. 1989;28(2):193-213.

© 2019 Whitaker RC et al. *JAMA Network Open.*
