# Supplementary material for: Effect of a Trauma-Awareness Course on Teachers’ Perceptions of Conflict With Preschool-Aged Children From Low-Income Urban Households: A Cluster Randomized Clinical Trial
Source: JAMA Netw Open. 2019 Apr 26;2(4):e193193. doi: 10.1001/jamanetworkopen.2019.3193 (PMC6487571; doi:10.1001/jamanetworkopen.2019.3193)
Supplement: Supplement 3. — Data Sharing Statement [file jamanetwopen-2-e193193-s003.pdf]

Data Sharing Statement

Whitaker. Effect of a Trauma-Awareness Course on Teachers' Perceptions of Conflict With Preschool-Aged Children From Low-Income Urban Households. *JAMA Netw Open*. Published April 26, 2019. 10.1001/jamanetworkopen.2019.3193

Data
Data available: No
